# Establishment of the CRISPR-Cpf1 gene editing system in *Bacillus licheniformis* and multiplexed gene knockout

**DOI:** 10.1016/j.synbio.2024.08.002

**Published:** 2024-08-08

**Authors:** Suxin Liu, Fengxu Xiao, Youran Li, Yupeng Zhang, Yanling Wang, Guiyang Shi

**Affiliations:** a Key Laboratory of Industrial Biotechnology, Ministry of Education, School of Biotechnology, Jiangnan University, Wuxi, 214122, PR China; b National Engineering Research Center for Cereal Fermentation and Food Biomanufacturing, Jiangnan University, 1800 Lihu Avenue, Wuxi, 214122, Jiangsu, PR China; c Jiangsu Provincial Engineering Research Center for Bioactive Product Processing, Jiangnan University, Wuxi, 214122, Jiangsu, PR China

**Keywords:** *Bacillus licheniformis*, Gene editing, CRISPR-Cpf1, MCherry

## Abstract

*Bacillus licheniformis* is a significant industrial microorganism. Traditional gene editing techniques relying on homologous recombination often exhibit low efficiency due to their reliance on resistance genes. Additionally, the established CRISPR gene editing technology, utilizing Cas9 endonuclease, faces challenges in achieving simultaneous knockout of multiple genes. To address this limitation, the CRISPR-Cpf1 system has been developed, enabling multiplexed gene editing across various microorganisms. Key to the efficient gene editing capability of this system is the rigorous screening of highly effective expression elements to achieve conditional expression of protein Cpf1. In this study, we employed mCherry as a reporter gene and harnessed P_*mal*_ for regulating the expression of Cpf1 to establish the CRISPR-Cpf1 gene editing system in *Bacillus licheniformis*. Our system achieved a 100 % knockout efficiency for the single gene *vpr* and up to 80 % for simultaneous knockout of the double genes *epr* and *mpr*. Furthermore, the culture of a series of protease-deficient strains revealed that the protease encoded by *aprE* contributed significantly to extracellular enzyme activity (approximately 80 %), whereas proteases encoded by *vpr*, *epr*, and *mpr* genes contributed to a smaller proportion of extracellular enzyme activity. These findings provide support for effective molecular modification and metabolic regulation in industrial organisms.

## 1 Introduction

*Bacillus licheniformis* is a Gram-positive facultative anaerobe commonly found in soil, known for its strong resistance, moderate growth rate, rich enzyme systems, and high enzyme production [[1], [2], [3], [4]]. It is a widely used industrial microbial strain. However, the lack of efficient gene editing tools and extremely low transformation efficiency greatly constrain molecular biology research on *B. licheniformis*, severely hindering the development of engineered strains and their application in industrial production [5].

Current gene editing methods for *B. licheniformis* are limited to temperature-sensitive plasmid knockout, linear fragment transformation, and CRISPR-Cas9 system knockout [[6], [7], [8]]. The temperature-sensitive plasmid knockout relies on the cell's own homologous double exchange to knock out the target gene, followed by recombinase removal of the resistance marker gene [6]; for linear fragment knockout, direct transformation of knockout cassette fragments utilizes the cell's own homologous recombination to replace the target gene [7]. Due to the extremely low endogenous recombination efficiency of *B. licheniformis*, both methods depend on extensive colony PCR to have a chance of selecting positive colonies [9]. Moreover, plasmid construction is complex and time-consuming, unable to perform simultaneous multigene knockouts, with very low editing efficiency, and even prone to resistance residue. Compared to the previous two gene editing systems, the CRISPR-Cas9 gene editing system has the advantages of high knockout efficiency, simple operation, and short cycle [10]. However, it also has many defects, such as the large relative molecular mass of Cas9 making transformation difficult, high off-target rate, and certain toxicity, which limit its application in *B. licheniformis* [11]. Therefore, there is an urgent need to develop a simple and efficient genome editing tool for *B. licheniformis*.

The introduction of Cpf1 has provided an alternative to the CRISPR toolkit [12]. Compared to the classic SpCas9, the CRISPR-associate protein and crRNA from *Francisella novicida* Cpf1 (FnCpf1) are shorter, and Cpf1 endonuclease requires only a single promoter to drive multiple crRNAs simultaneously [13,14]. The key components of the CRISPR-Cpf1 system include the Cpf1 protein, crRNA, and the provided homology-directed repair (HDR) template. The crRNA guides the Cpf1 protein to the target site; Cpf1 is responsible for cutting the target DNA, resulting in a double-stranded break (DSB) [15]. Non-homologous end joining (NHEJ) and homologous recombination (HR) are two mechanisms for repairing DSBs in the CRISPR mechanism, with the latter being predominantly used in bacteria [9,16]. The homology repair template allows us to produce specific gene knockout fragments via polymerase chain reaction, not only enabling more efficient screening of gene knockout strains but also improving genome stability [17]. Yang found that FnCpf1 is less toxic than Cas9 and achieved a 100 % editing efficiency in *Corynebacterium glutamicum* [18]. Cui conducted knockout validation in *Bacillus subtilis* and concluded that the functionality of CRISPR-Cpf1 in single-gene knockouts exceeded that of the same strategy using CRISPR-Cas9, with the editing efficiency also reaching 100 % [13]. To date, the CRISPR-Cpf1 system has been used as an efficient genomic editing tool in various biological systems [13,[18], [19], [20], [21], [22], [23], [24]], yet a *B*. *licheniformis* gene editing method based on the CRISPR-Cpf1 system has not yet been established.

Introducing the CRISPR-Cpf1 system into *B*. *licheniformis* presents a significant challenge due to the inherently low transformation efficiency of recombinant vectors, which is a natural characteristic of the bacterium [8]. The size of the vector and the expression of the carried genes are two main factors that influence this process; the former affects substance transport across membranes, while the latter hinders cell growth and reproduction [10]. To mitigate the negative impact of vector size, we constructed a CRISPR-Cpf1 gene knockout vector using the small pJOE8999 plasmid as a backbone. Fine-tuning the expression level of Cas proteins remains a key aspect of applying the CRISPR-Cpf1 system [10]. Liu believes that, compared to constitutive expression systems, inducible expression systems are beneficial for dynamic gene regulation and effectively prevent the impact of Cas protein expression on host cell growth [25]. To reduce the strong toxicity of nucleases on host cells, Wang chose the lactose-inducible promoter P_*lac*_ to drive Cpf1 expression in *Clostridium sporogenes* [24]. Therefore, inducible expression systems can minimize the toxicity caused by Cpf1 gene expression. The xylose-induced promoter and mannitol-induced promoter are the most commonly used inducible promoters in *B*. *licheniformis* [8,26]. The xylose-induced expression system is strictly controlled by the repressor protein encoded by *xylR*, offering high expression levels but at a high cost. Additionally, the xylose isomerase regulon contains *xylR* and P_*xyl*_ elements spanning 1297bp, inevitably resulting in oversized recombinant plasmids that affect transformation [27,28]. The mannitol-inducible expression system uses a cheap and stable carbon source as an inducer, with the advantages of being efficient and non-toxic. However, studies have shown that the mannitol promoter has basal leakage, and furthermore, the recovery medium contains mannitol and sorbitol, which could directly induce expression leading to transformation failure or even cell death [26]. There is an urgent need to develop a stringent inducible promoter to regulate the expression of the Cpf1 gene in *B*. *licheniformis*. In our study, we used red fluorescent protein to verify the stringency of the maltose-inducible expression system. Maltose serves as an inducer with low industrial cost and is safe and non-toxic, making it an ideal choice for regulating expression systems [29]. We utilized the pJOE8999 vector as a backbone and harnessed the maltose promoter P_*mal*_ to construct a CRISPR-Cpf1 gene editing system, providing support for effective molecular modification and metabolic regulation in *B*. *licheniformis* and offering prospects for genetic modification in other industrially relevant *Bacillus* strains.

## 2 Materials and methods

### 2.1 Bacterial strains, plasmids, and culture conditions

The bacterial strains, plasmids, and primers used in this study are listed in Table S1. Table S2 lists the primers used in the experiment and the primers designed. A series of recombinant strains were constructed using *B*. *licheniformis* CICIM B1391 as the parent strain, while *E*. *coli* JM109 was used for constructing recombinant vectors.

The electroporation of *B. licheniformis* was conducted with reference to the method described by Xiao [30]. Both *B*. *licheniformis* and *E*. *coli* were cultured in LB medium (1 % tryptone, 0.5 % yeast extract, 1 % NaCl, pH 7.2). Antibiotics (kanamycin at 30 μg/mL, tetracycline at 20 μg/mL, and ampicillin at 100 μg/mL; purchased from Sigma) were added to the medium as needed. Unless otherwise specified, the culture temperature was maintained at 37 °C with a shaking speed of 250 rpm.

The medium used for the production of alkaline protease (fermentation medium: 3 % sucrose, 0.912 % K_2_HPO_4_⋅3H_2_O, 0.136 % KH_2_PO_4_, 1 % (NH_4_)_2_HPO_4_, 0.05 % FeCl_3_, 1 % tryptone, 0.5 % yeast powder, 0.2 % corn steep liquor dry powder, 5 % liquid nitrogen source, pH 7.2)

### 2.2 Plasmid construction

Gene fragments were amplified and identified using 2 × Phanta Max Master Mix polymerase (Vazyme Biotech, Nanjing, China) and 2 × Taq PCR Master Mix polymerase (TaKaRa, Hangzhou, China). Plasmids were assembled using fast restriction enzymes and T_4_ DNA ligase (Thermo Fisher Scientific, USA). The constructed recombinant plasmids were confirmed by sequencing (Sangon Biotech, Shanghai, China), and all primers and single-stranded DNA were synthesized by Sangon Biotech (Shanghai, China).

#### 2.2.1 Construction of maltose-inducible expression vector

The shuttle vector pHY served as the backbone for constructing the maltose-inducible expression vector. Using chromosomal DNA from *B*. *licheniformis* CICIM B1391 as a template, the maltose promoter (P_*mal*_) fragment was amplified by PCR with primers P_*mal*_-*Bgl*II-F and P_*mal*_-*Xho*I-R. The pHY-eGFP vector was digested with restriction enzymes *Bgl*II and *Xho*I to obtain a linearized pHY-eGFP vector, into which the P_*mal*_ fragment was cloned by homologous recombination, resulting in the pHY-P_*mal*_-eGFP plasmid.

The mCherry fragment synthesized by the company had 15 bp homologous arms on both ends. The eGFP gene fragment was excised from pHY-P_*mal*_-eGFP using restriction enzymes *Xho*I and *Sal*I to obtain a linearized pHY-P_*mal*_ vector. The mCherry fragment was cloned into the linearized pHY-P_*mal*_ vector by homologous recombination, and the plasmid pHY-P_*mal*_-mCherry was extracted.

#### 2.2.2 Construction of *vpr* gene knockout vector

The gene encoding the protease *vpr* in *B*. *licheniformis* CICIM B1391 (CP005965 REGION: 4022002–4024422) was selected as the target gene for knockout experiments. The shuttle vector pJOE8999 served as the backbone for constructing the pJOE8999-P_*mal*_-Cpf1-*vpr* recombinant plasmid.

Using chromosomal DNA from *B*. *licheniformis* as a template, the P_*mal*_ fragment was amplified by PCR with primer pair P_*mal*_-F/P_*mal*_-R. The Cpf1 gene fragment was amplified from a synthetic Cpf1 gene template using primer pair Cpf1-F/Cpf1-R. The P_*mal*_-Cpf1 fragment was obtained through overlapping PCR using P_*mal*_ and Cpf1 fragments as templates with primer pair P_*mal*_-F/Cpf1-R. Using a vector carrying the *amyL* terminator from the laboratory as a template, the *amyL* terminator fragment was amplified by PCR with primer pair ter-F/ter-R. Finally, using primer pair P_*mal*_-F/ter-R, the Cpf1 expression cassette was obtained through another round of overlapping PCR using P_*mal*_-Cpf1 and *amyL* terminator fragments as templates. Homologous recombination was performed between the expression cassette and the pJOE8999 shuttle vector digested with *Kpn*I/*Bsr*GI to obtain the pJOE8999-P_*mal*_-Cpf1 recombinant plasmid. The three PN2-mediated crRNA expression frames were synthesized by Shanghai Biotechnology, and were used as templates for PCR amplification with the primers crRNA-F/crRNA-R and homologous recombination with the *Sma*I/*Eco*RI digested pJOE8999-P_*mal*_-Cpf1 plasmid to obtain the three pJOE8999-P_*mal*_-Cpf1-crRNA plasmids. Among them, the location choices of the three targets are described in Table S1.

Using the *B*. *licheniformis* genome as a template, PCR amplification was performed with primer pairs *vpr*-Left-F/*vpr*-Left-R and *vpr*-Right-F/*vpr*-Right-R to obtain the left and right homologous arm sequences of the *vpr* gene, respectively. Then, using the left and right homologous arm fragments as templates, overlapping PCR amplification was carried out with primer pair *vpr*-Left-F/*vpr*-Right-R to obtain the left-right homologous arm sequence. This sequence was ligated to three pJOE8999-P_*mal*_-Cpf1-crRNA plasmids double digested by *Sma*I/*Bam*HI to obtain three *vpr* knockout plasmids, named pJOEC1, pJOEC2, and pJOEC3, respectively.

#### 2.2.3 Construction of *mpr*-*epr* gene knockout vector

The genes encoding *mpr* protease (CP005965 REGION: 338171–339112) and *epr* protease (CP005965 REGION: 1221255–1223030) from *B*. *licheniformis* CICIM B1391 were selected as targets for the double knockout experiment. Using the knockout plasmid pJOE8999-P_*mal*_-Cpf1-*vpr-*1 as a template, three fragment PCRs were performed with primer trios *mpr*-e*pr*-crRNA-F1/*mpr*-e*pr*-crRNA-R1, *mpr*-e*pr*-crRNA-F2/*mpr*-e*pr*-crRNA-R2, and *mpr*-*epr*-crRNA-F3/*mpr*-*epr*-crRNA-R3 to obtain three fragments named *mpr*-*epr*-crRNA1, *mpr*-*epr*-crRNA2, and *mpr*-*epr*-crRNA3. Subsequently, overlapping PCR amplification was performed with *mpr*-*epr*-crRNA1 and *mpr*-*epr*-crRNA2, *mpr*-*epr*-crRNA2 and *mpr*-*epr*-crRNA3 using primer pairs *mpr*-*epr*-crRNA-F1/*mpr*-*epr*-crRNA-R2 and *mpr*-*epr*-crRNA-F2/*mpr*-*epr*-crRNA-R3 to obtain two fragments named *mpr*-*epr*-crRNA1-*mpr*-*epr*-crRNA2 and *mpr*-*epr*-crRNA2-*mpr*-*epr*-crRNA3. Finally, using these two fragments as templates, overlap extension PCR amplification was carried out with primer pair *mpr*-*epr*-crRNA-F1/*mpr*-*epr*-crRNA-R3 to obtain the final fragment *mpr*-*epr*-crRNA. This fragment was ligated with the pJOE8999-P_*mal*_-Cpf1-*vpr-*1 plasmid digested with *Eco*RI/*Sma*I to complete the crRNA replacement, preparing for the next step of homologous arm replacement. Next, the homologous arms were assembled. Using the *B*. *licheniformis* genome as a template, PCR amplification was performed with primer pairs *epr*-Left-F/*epr*-Left-R and *epr*-Right-F/*epr*-Right-R to obtain the left and right homologous arm sequences of the *epr* gene. Then, using these two fragments as templates, overlapping PCR amplification was carried out with primer pair *epr*-Left-F/*epr*-Right-R to obtain the *epr* homologous arm sequence. Similarly, PCR amplification was performed with primer pairs *mpr*-Left-F/*mpr*-Left-R and *mpr*-Right-F/*mpr*-Right-R to obtain the left and right homologous arm sequences of the *mpr* gene. Then, using these two fragments as templates, overlapping PCR amplification was carried out with primer pair *mpr*-Left-F/*mpr*-Right-R to obtain the *mpr* homologous arm sequence. Finally, using the *epr* and *mpr* homologous arm sequences as templates, overlap extension PCR amplification was performed with primer pair *epr*-Left-F/*mpr*-Right-R to obtain the *epr*-*mpr* homologous arm sequence fragment. This fragment was ligated with the prepared plasmid digested with *Sma*I/*Kpn*I to complete the replacement of homologous arms, ultimately obtaining the pJOE8999-P_*mal*_-Cpf1-*epr*-*mpr* double knockout vector and named pJOECEM.

### 2.3 Genome editing of *Bacillus licheniformis* using the CRISPR-Cpf1 system

The constructed knockout vectors pJOEC1, pJOEC2, pJOEC3 (listed in Table S1) were electroporated into competent *B*. *licheniformis* cells, resulting in recombinant strains BLCP1, BLCP2, BLCP3 [30]. These recombinant strains were cultured in LB medium supplemented with kanamycin at 37 °C and 250 rpm. When the cells were grown for 6 h and the OD_600_ reached about 4, a 1.5 % concentration of maltose inducer was added to activate the expression of Cpf1 protein. After an additional 24 h of incubation, colony PCR verification was performed using the primer pair *vpr*-qiaochu-yz-F/*vpr*-qiaochu-yz-R, followed by DNA sequencing of the knockout strains. The same method was applied to achieve knockout of the *mpr* and *epr* genes. The knockout efficiency was evaluated by randomly selecting transformants from each transformation, and the knockout strains were identified by polymerase chain reaction, gel electrophoresis and Sanger sequencing [5]. The gene editing efficiency is calculated as the percentage of knockout strains to the total number of strains.

### 2.4 Elimination of knockout plasmids

Since pE194 cannot replicate at high temperatures, to eliminate the gene knockout plasmid from positive strains, single colonies were inoculated into LB medium without antibiotics at 37 °C (250 rpm) [31]. If necessary, 1.5 % maltose was added to enhance plasmid elimination efficiency. After scribing on a flat plate, the cultures were spread onto antibiotic-free plates. Single colonies grown on these plates were screened on agar plates with and without kanamycin. Colonies sensitive to kanamycin indicated successful plasmid loss and were named BL_2_Δ*vpr* and BL_2_Δ*epr*-*mpr*, respectively.

### 2.5 Determination of biomass and alkaline protease activity

The strains BL_2_, BL_2_Δ*vpr*, and BL_2_Δ*epr*-*mpr* were cultured overnight for approximately 16 h until the OD_600_ reached 4.0–4.5. A 3 % inoculum was then transferred to fermentation medium and incubated at 37 °C with shaking at 250 rpm. Samples were collected after 24h, 48h, and 72h of fermentation to determine OD_600_ and enzyme activity. An Ultrospec 3000 spectrophotometer (Pharmacia Biotech, Piscataway, NJ, USA) was used to monitor biomass by measuring OD_600_, diluting cell culture samples to an appropriate concentration prior to detection. Protease activity was measured using the Folin method, with some modifications based on practical conditions: casein was pre-wetted with NaOH and dissolved in phosphate buffer during a 30-min boiling water bath; diluted enzyme solution (1 mL) was mixed with casein solution (1 mL), and the reaction at 40 °C was stopped after 10 min by adding 2 mL TCA [32]. One unit of enzyme activity (U) is defined as the amount of enzyme required to produce 1 μg molecule of tyrosine per minute from casein hydrolysis at 40 °C and pH = 10.5.

### 2.6 Statistical analysis methods

All experiments were independently repeated three times, and the average value was taken as the final result. The differences between two sets of data were analyzed using a 2-tailed Student's t-test, while the differences between multiple sets of data were compared using one-way ANOVA and Tukey's test. "*" and "* * *" were used to indicate the significance of p < 0.05 and p < 0.001, respectively.

## 3 Results and discussion

### 3.1 The red fluorescent protein mCherry is superior to green fluorescent protein eGFP

Fluorescent proteins are often used as reporter genes due to their stability, high sensitivity, and ease of detection, allowing for the measurement of transcriptional expression characteristics of upstream promoters. Compared to the classic green fluorescent protein eGFP, red fluorescent proteins have a broader range of excitation and emission wavelengths, lower cytotoxicity, and variants like mCherry exhibit better maturation and excellent photostability with lower background levels [33].

**Fig. 1:**
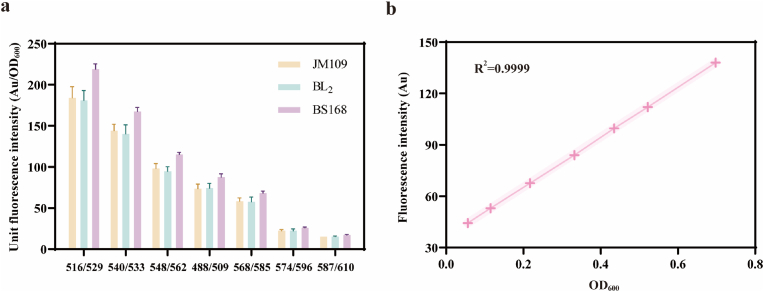
**The fluorescence intensity of mCherry was analyzed using primitive bacteria.** (a) The fluorescence intensities produced by the three primitive bacteria under different fluorescence detection conditions, where 516/529 denotes the peak excitation and peak emission wavelengths of YFP; 540/533 denotes the peak excitation and peak emission wavelengths of mBannana; 548/562 denotes the peak excitation and peak emission wavelengths of mOrange; 488/509 denotes the peak excitation and peak emission wavelengths of eGFP; and 488/509 denotes the peak excitation and peak emission wavelengths of eGFP; 568/585 for peak excitation and peak emission wavelengths of mTangerine; 574/596 for peak excitation and peak emission wavelengths of mStrawberry; and 587/610 for peak excitation and peak emission wavelengths of mCherry. (The average of three repetitions, and the error bar corresponds to a standard deviation). (b) Linear relationship analysis of the fluorescence intensity of mCherry with OD_600_.

*E. coli*, *B. licheniformis*, and *B. subtilis* were each inoculated into 15 mL LB and fermented for 24 h before sampling. The samples were washed twice with PBS buffer, diluted to an appropriate concentration, and the OD_600_ and fluorescence values were measured. The fluorescence exhibited by the three bacterial strains under different excitation and emission wavelengths was analyzed. As shown in Fig. 1a, the baseline fluorescence values of the three original strains for different fluorescences were almost identical. Under the detection conditions for the red fluorescent protein mCherry, the baseline fluorescence values produced were the lowest at only 15, which is approximately 1/13th of the fluorescence value produced under YFP detection conditions and 1/5th of that under eGFP detection conditions. This indicates that fluorescent proteins are universally applicable to different bacterial species, and the results show that mCherry has excellent photostability and the lowest background.

Subsequently, the recombinant strain BLPM was inoculated into 15 mL LB and cultured for 24 h. The sample was diluted to a certain multiple to determine the linear range of fluorescence values and OD_600_. As shown in Fig. 1b, there is a linear response relationship when OD_600_ is within the range of 0.056–0.698, with the best linear correlation between fluorescence values and cell density of the bacteria. Fluorescence detection settings for the microplate reader: shake the microplate for 10s, excitation wavelength at 587 nm, absorbance at 610 nm, gain value at 120.

### 3.2 Exploration and identification of maltose promoters

Promoters, as a crucial component of expression systems, play a key role in regulating gene expression levels. To better regulate the expression of Cpf1, we mined maltose promoters from the maltose operon, which mainly regulates maltose metabolism. The operon consists of three functional genes: *malA*, *malR*, *malP* [34]. *B. subtilis* can utilize maltose and has increased the activity of the P_*mal*_ through site-directed mutagenesis [29]. Wen and others have developed a maltose-inducible expression system based on the P_*mal*_ in *B. subtilis*, using GFP as a reporter gene to confirm the effectiveness of this expression system [35]. At the amino acid level, metabolic pathways, and enzyme production, *B. licheniformis* shares high similarity with *B. subtilis* [36]. An expression system based on P_*mal*_ has not yet been constructed in *B. licheniformis*, suggesting potential applications for this inducible expression system in *B. licheniformis*. Using the red fluorescent protein gene (mCherry) as a reporter gene, we evaluated the inducible regulatory performance of the P_*mal*_ promoter in *B. licheniformis* by adding corresponding inducers.

### 3.3 Fluorescence intensity affected by different carbon sources

**Fig. 2:**
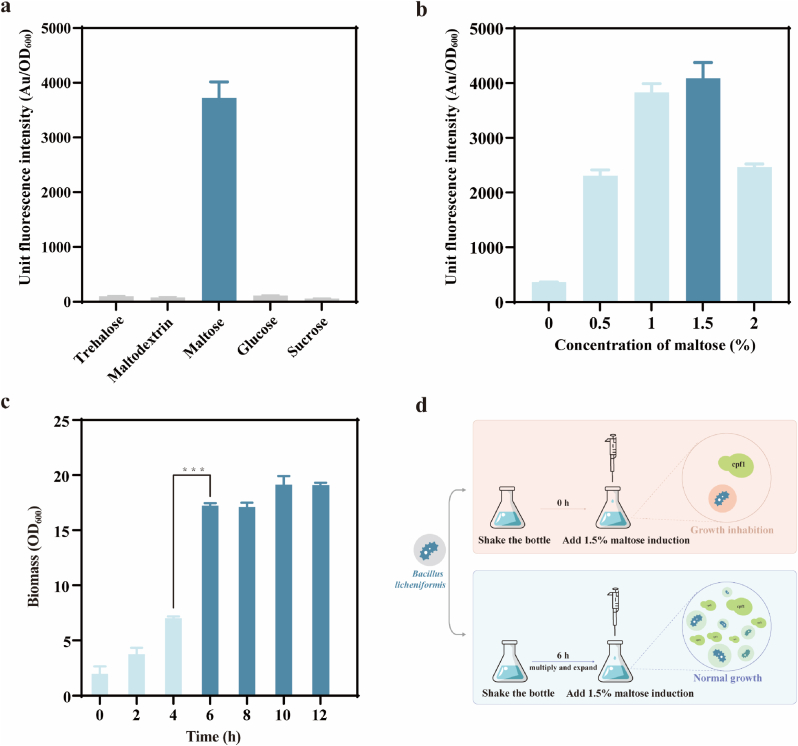
**Expression of mCherry under the control of mal promoter.** (a) Effect of different carbon sources on the strength of the mal promoter in the mCherry reporter assay. (b) Effect of different concentrations of maltose on the strength of the mal promoter in the mCherry reporter assay. (c) The growth status of the strain under different maltose induced conditions (d) Strain growth was inhibited by adding maltose at 0 h and normalized by adding maltose at 6 h.

Firstly, the fluorescence intensity under the influence of different carbon sources was tested. The recombinant strain BLPM was inoculated into LB medium supplemented with 1.5 % maltose, 1.5 % maltodextrin, 1.5 % trehalose, 1.5 % glucose, and 1.5 % sucrose, respectively. A single colony of BLP was inoculated into LB medium as a blank control. After 24 h of fermentation, samples were taken, washed twice with PBS buffer, diluted, and then the mCherry fluorescence intensity and OD_600_ values were measured. As shown in Fig. 2a, the fluorescence value was 3727 AU/OD_600_ when maltose was added. When equal concentrations of trehalose, maltodextrin, glucose, and sucrose were added, the detected fluorescence values were 102, 78, 115, and 58 AU/OD_600_, respectively, indicating a clear maltose-induced characteristic in the strain. We also tested the performance of the xylose promoter under the same conditions and found that the maltose promoter was more rigorous, and the relevant proof is described in detail in the supporting information. Furthermore, the fluorescence values produced by different concentrations of maltose were tested by inoculating single colonies of BLPM into media with final maltose concentrations of 0 %, 0.5 %, 1 %, 1.5 %, and 2 %, and measuring mCherry fluorescence intensity and OD_600_ values. As shown in Fig. 2b, the unit fluorescence intensity of the mCherry protein gene regulated by the promoter P_*mal*_ increased with the concentration of maltose before decreasing, suggesting that the expression level of the target gene could be regulated by the concentration of maltose. Moreover, when the concentration of maltose was 1.5 %, the induction effect was optimal. In order to investigate the impact of maltose addition time on recombinant strain growth, we conducted a series of experiments wherein maltose was added at varying time points. The results of these experiments are presented in Fig. 2c. Notably, the growth of the recombinant strains was inhibited by as much as 88.24 % when maltose was added at 0 h. On the other hand, it is worth noting that we found that the growth of *Bacillus licheniformis* wild bacteria was also significantly inhibited when maltose was added at 0 h. This particular phenomenon has been reported in *Saccharomyces cerevisiae* [37,38], which also reveals that the cells should be given sufficient time to grow when the inducer is added. Therefore, we chose to add maltose for 6 h in order to minimize its effect (Fig. 2d). In summary, the results indicate that the maltose-inducible expression system is suitable for producing heterologous proteins in *B*. *licheniformis* and that maltose is an ideal effector for initiating Cpf1 nuclease.

### 3.4 1.5 % maltose induction for Cpf1 expression used in genome editing

**Fig. 3:**
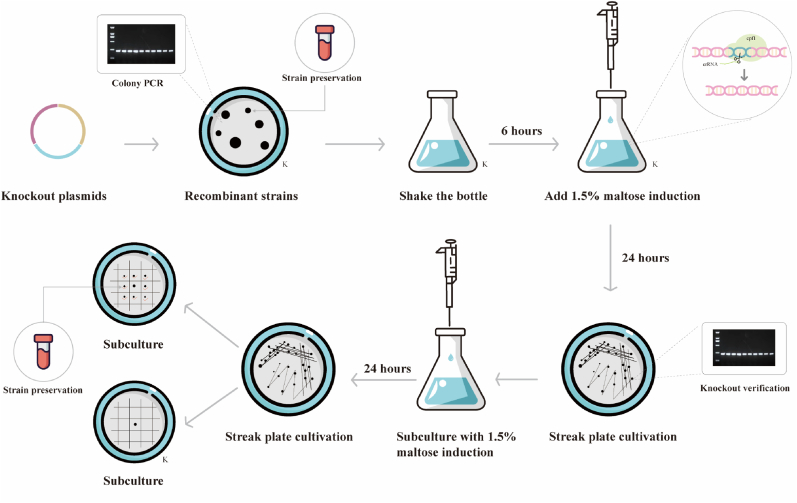
**Overview of CRISPR-Cpf1 gene editing methods established in *Bacillus licheniformis*.** The letter K in the lower right corner indicates that the medium contains kanamycin resistance.

Although the CRISPR-Cpf1 system has been used for genome editing in various organisms, there have been no reports on its use in *B*. *licheniformis* [39]. Here, we attempted to use plasmid pJOE8999-P_*mal*_-Cpf1-*vpr* to knock out the *vpr* gene in *B*. *licheniformis*. For gene editing, the first step is to design specific target sites. With the help of homologous repair templates, the identified targets can be modified or removed, and specific sequences can be introduced into the genome to achieve precise genome editing. In order to cleave the *vpr* gene, we designed three sgRNA targets and obtained three recombinant strains, named BLC1, BLC2, and BLC3, respectively (listed in Table S1). The Cpf1-mediated genome editing process induced by maltose can be divided into three main steps, as shown in Fig. 3. First, recombinant strains BLC1, BLC2, and BLC3 were cultured at 37 °C and 250 rpm for 6 h. During this period, the recombinant strains proliferated extensively and the recombinant plasmids replicated in large quantities. Then, 1.5 % maltose was added to the medium to induce Cpf1 expression, triggering DNA double-strand breaks and homologous recombination repair; after an additional 24 h of culture, lines were streaked on solid medium containing 30 μg/mL kanamycin to form single colonies, which were then verified by colony PCR to confirm successful *vpr* gene knockout. Finally, colonies with successful knockouts were inoculated into LB medium and cultured at 37 °C (250 rpm) (if necessary, 1.5 % maltose could be added to improve plasmid elimination efficiency), streaked on non-antibiotic plates, and then single colonies grown were screened on two agar plates with or without kanamycin to identify strains that had successfully lost the plasmid.

**Fig. 4:**
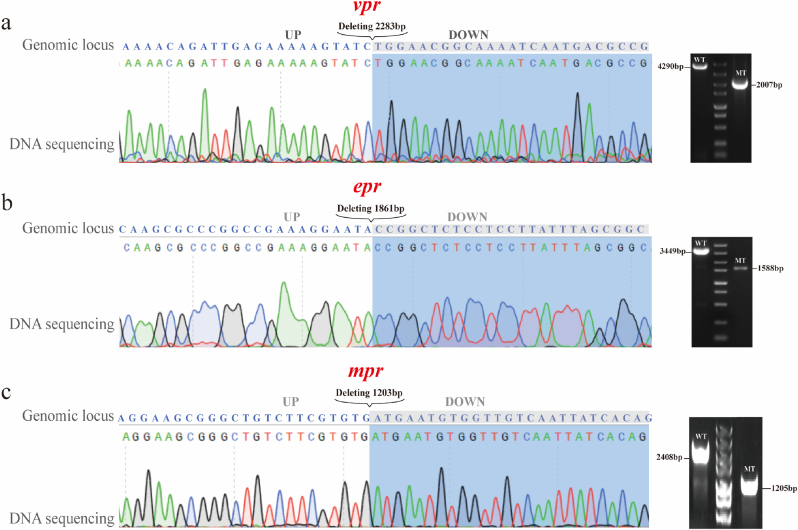
**Confirmation of disruption of *vpr*, *epr* and *mpr*. The DNA sequencing, alignment result of the deletion-carrying mutant and agarose gel electrophoresis.** (a) *vpr* gene deletion, losing 2283 bp. (b) *epr* gene deletion, losing 1861 bp. (c) *mpr* gene deletion, losing 1203 bp.

Three recombinant strains used for *vpr* gene knockout were induced and cultured, in general, gene-deficient strains can be obtained by colony PCR validation of the transformants, and the knockout efficiency is the number of gene-deficient strains obtained divided by the total number of strains [15,40]. Theoretically, only strains with successful knockout can survive and form single colonies visible to the naked eye. In practice, the number of single colonies on the plate was found to be low, with only a few colonies observed following the induction and strip culture process. In the *vpr* knockout experiment, only 20 transformants were obtained, and the relevant images are presented in the supplementary materials, colony PCR was performed using the primers *vpr*-qiaochu-yz-F and *vpr*-qiaochu-yz-R. Successful gene knockout strains produced a 2007 bp band, while strains with unsuccessful knockouts retained the original 4290 bp band from the wild-type genome (Fig. 4a). The colony PCR results showed that the target gene disruption efficiency for pJOEC1, pJOEC2, and pJOEC3 was 100 %, indicating that the CRISPR-Cpf1 gene editing system was successfully constructed in *B*. *licheniformis* and could efficiently knock out the *vpr* gene.

The same procedure was followed for the dual gene knockout of *epr* and *mpr* resulted in 10 transformants. The knockout efficiency of *epr* was verified using the primers *epr*-qiaochu-yz-F and *epr*-qiaochu-yz-R, with successful knockouts producing a 1588 bp band, and unsuccessful ones retaining the original 3449 bp band from the wild-type genome (Fig. 4b). Similarly, *mpr* knockout efficiency was verified using the primers *mpr*-qiaochu-yz-F and *mpr*-qiaochu-yz-R, with successful knockouts producing a 1205 bp band, and unsuccessful ones retaining the original 2408 bp band from the wild-type genome (Fig. 4c). The results showed that for plasmids designed for dual gene knockouts, the disruption efficiency was 100 % for *epr* and 80 % for *mpr*, indicating that the efficiency of the CRISPR-Cpf1 dual gene editing system was lower than that of the single gene editing system in *B*. *licheniformis*.

### 3.5 Characteristics of gene-edited strains

*Bacillus* species secrete a variety of extracellular proteases, which can degrade extracellular proteins to varying degrees depending on their folding state and proteolytic sensitivity [41]. Therefore, the presence of extracellular proteases hinders the expression of heterologous secreted proteins, increasing the difficulty of industrial production in *Bacillus* [42]. Studies have shown that knocking out extracellular protease genes in *Bacillus subtilis* can significantly increase the yield of various enzymes including xylanase, *α*-amylase, and endoglucanase [43]. By suppressing protease expression, the yield of *α*-amylase in *B*. *licheniformis* increased by 250 % [41]. Serine proteases and metalloproteases are among the extracellular proteases produced by *Bacillus licheniformis* [36]. Therefore, we attempted to knock out the alkaline protease gene *vpr* as our first target to verify knockout efficiency. Subsequently, we knocked out the alkaline protease genes *epr* and *mpr* to obtain chassis strains suitable for the expression of exogenous proteins.

**Fig. 5:**
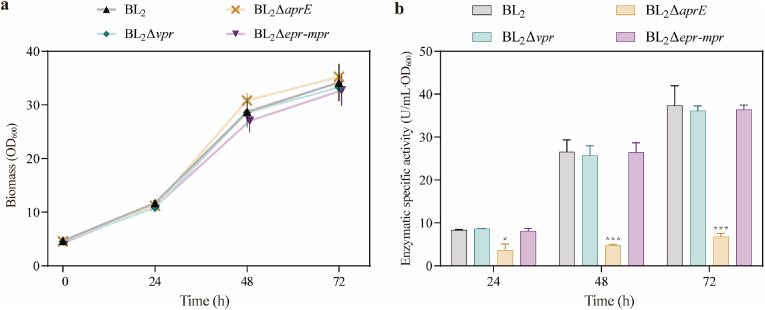
**Growth of the original strain BL**_**2**_**and knockout strains BL**_**2**_**Δ*vpr*, BL**_**2**_**Δ*epr*-*mpr* and BL**_**2**_**Δ*aprE* of Bacillus licheniformis and their protease activities after fermentation.** (a) Growth and alkaline protease activity of BL_2_ and BL_2_Δ*vpr*, BL_2_Δ*epr*-*mpr* and BL_2_Δ*aprE*. (b) The alkaline protease activities of BL_2_ and BL_2_Δ*vpr*, BL_2_Δ*epr*-*mpr* and BL_2_Δ*aprE*, Bars with "*" and "* * *" indicate p < 0.05 and 0.001 relative to the original strain, respectively.

Through molecular-level verification, we successfully obtained gene knockout strains BL_2_Δ*vpr* and BL_2_Δ*epr*-*mpr*. We measured the growth curves of these strains (Fig. 5a) and also tested our laboratory-preserved BL_2_Δ*aprE* strain, which was obtained through a temperature-sensitive plasmid combined with the FLP/FRT recombination system [44]. The OD_600_ at 72 h of growth showed that the growth rates of knockout strains BL_2_Δ*vpr*, BL_2_Δ*epr*-*mpr*, and BL_2_Δ*aprE* were essentially consistent with that of the wild-type strain. This result indicates that disruption of the *vpr*, *epr*, *mpr*, and *aprE* genes did not have a significant negative impact on bacterial growth.

To further validate the knockout effects, the specific activities of alkaline proteases from the four strains were measured at pH 10.5 after 24h, 48h, and 72h of cultivation, as shown in Fig. 5b. As expected, the specific activity values of alkaline proteases in the three knockout strains decreased to some extent. Specifically, the strains BL_2_Δ*vpr* and BL_2_Δ*epr*-*mpr* showed a slight reduction in enzyme activity compared to the original strain, but not a significant difference. In contrast, the BL_2_Δ*aprE* strain exhibited a dramatic decline in enzyme activity, reaching only 43.95 %, 18.02 %, and 18.22 % of the original strain's activity after 24h, 48h, and 72h, respectively, indicating a significant difference. These results suggest that the proteases *vpr*, *epr*, and *mpr* that we knocked out are not the main alkaline proteases in *B*. *licheniformis*, whereas *aprE* has high extracellular protease activity in this species, consistent with published data [45,46]. In *Bacillus* species, eight extracellular proteases play a major role: *aprE*, *bpr*, *epr*, *mpr*, *nprB*, *nprE*, *vpr*, and *wprA*; among them, *aprE*, *bpr*, *epr*, and *vpr* are alkaline proteases, with *aprE* and *nprE* accounting for the largest proportion of total extracellular proteolytic activity [47]. In summary, through molecular-level validation and enzyme production level verification, the knockout effects were confirmed. This demonstrates that the CRISPR-Cpf1 gene editing system is suitable for gene editing in *B*. *licheniformis* and represents a highly promising gene editing tool.

## 4 Discussion

Gene editing is an essential tool for molecular modification, and the advent of the third-generation gene editing tool, the CRISPR system, has propelled the rapid development of gene editing technologies [48]. In previous studies, researchers developed a CRISPR-Cas9 mediated genome editing tool in *B*. *licheniformis*, and Li constructed an integrated CRISPR/Cas9 expression vector targeting the *α-amyL* gene of *B. licheniformis*, with editing efficiencies at three target sites of 64.3 %, 70.9 %, and 47.1 % [8]. The knockout efficiency was less than 80 %, and the knockout plasmid was as long as 10880 bp, which is not conducive to transformation; Lu performed single-gene knockouts in *B. licheniformis* based on CRISPR-Cas9, with knockout efficiencies for *uprT*, *amyL*, and *chiA* genes of 97.3 %, 90 %, and 90 %, respectively, exceeding 90 % efficiency. However, their knockout plasmid system was too large, for example, the pWH-Δ*upp* plasmid was 13488 bp long, which exacerbated the difficulty of transformation [15]. None of these proved whether the system could accurately perform multi-gene editing. Chen integrated CRISPR-Cas9 nickase into the genome of *B. licheniformis*, achieving a knockout efficiency of 100 % for *yvmC* and a simultaneous double-gene knockout efficiency of 11.6 % for *epr* and *wprA*, indicating a disadvantage of lower multi-gene editing efficiency [5]. Subsequently, researchers developed the CRISPR-Cpf1 gene editing technology and made breakthrough progress. Wang Yi used the CRISPR-Cpf1 system to successfully delete two genes, *spo0A* and *pta*, in *Clostridium beijerinckii* with an editing efficiency of 100 % [24]. Liu achieved a double-gene knockout in B*acillus subtilis* with 100 % efficiency using CRISPR-Cpf1 [49]. Liang used the CRISPR-Cpf1 system in *Pichia pastoris*, achieving single-gene disruption rates of about 99 % and double-gene editing efficiencies of about 80 % [50]. CRISPR-Cpf1 demonstrated high editing efficiencies in both single and double-gene knockouts. In this study, we introduced the CRISPR-Cpf1 gene editing system into *B. licheniformis* for the first time and compared to the CRISPR-Cas9 system, our newly constructed editing system has several advantages. Firstly, our single-gene knockout efficiency is higher, achieving up to 100 % for the *vpr* gene deletion. Secondly, we validated multi-gene knockouts with good results, reaching up to 80 % efficiency for simultaneous *epr* and *mpr* double-gene deletions. Lastly, our plasmid is smaller; the *vpr* gene knockout plasmid is only 8424bp, reducing transformation stress. This system provides support for effective molecular modification and metabolic regulation in *B. licheniformis* and offers application prospects for genetic modification in other industrially relevant *Bacillus* species.

As an important component of the expression system, promoters play a key role in regulating gene expression levels [51]. Compared to constitutive promoters, inducible expression systems have the unique advantage of being able to artificially control the initiation or shutdown of gene transcription, separating bacterial growth from exogenous gene expression processes, reducing early-stage bacterial burden while promoting later-stage product accumulation [[52], [53], [54]]. In recent years, various types of inducible promoters have been developed and applied in metabolic regulation. Commonly used inducible promoters include the xylose-inducible promoter P_*xyl*_, sucrose-inducible promoter P_*sacB*_, and IPTG-inducible promoter P_*grac*_ [52]. The xylose-inducible expression system is characterized by high specificity, stringency, and safety [55,56]. Studies have shown that sucrose-induced expression of green fluorescent protein is significantly lower than that induced by xylose, and the stringency of sucrose promoters is not high [57]. IPTG concentration significantly affects protein expression levels and is a strong inducer; however, there is leaky expression in the absence of IPTG [58,59]. The strength of xylose-inducible promoters and sucrose-inducible promoters still needs to be improved, while the high cost of xylose and IPTG and safety concerns about IPTG hinder the widespread application of related expression systems. The maltose promoter shows advantages [29,[34], [35], [36]]; using mCherry as a reporter gene, we explored and identified the stringency of the inducible promoter P_*mal*_; a 1.5 % inducer can achieve efficient expression of fluorescent proteins. Expressing exogenous genes with maltose promoters has advantages such as low cost and safety of the inducer maltose, high induction strength, and good controllability, making it particularly suitable for industrial production applications.

A rich array of proteases is one of the main characteristics of the *Bacillus* genus, with these enzymes located in the cytoplasm, cell membrane, cell wall, and external environment [46]. They assist in acquiring peptides and amino acids from organic matter and play a significant role in spore formation [60,61]. With advancing research, eight extracellular proteases have been identified, including *aprE*, *bpr*, *epr*, *vpr*, and *wprA*, which belong to the serine protease family, and *mpr*, *nprB*, and *nprE*, which are metalloproteases [[62], [63], [64], [65], [66], [67], [68], [69]]. Fourteen proteases have been identified within the cytoplasm of vegetative cells, where they are active, indicating a more complex classification of intracellular proteases [46]. Precise knockout of genes encoding these proteases is essential for revealing their functions. Researchers constructed strain DB104 by knocking out the *aprE* and *nprE* genes using the native recombination system and found that the extracellular protease activity was only 4 % of the wild type, but spores still formed normally, indicating that neither *aprE* nor *nprE* proteases are essential for cell growth and spore formation [69]. Further construction of strain WB600 (Δ*aprE*, Δ*bpr*, Δ*epr*, Δ*mpr*, Δ*nprB*, Δ*nprE*) revealed that after 24 h of culture, the extracellular protease activity was 0.32 % of the wild type. However, as the culture time increased, the activity gradually rose, suggesting the presence of other uncharacterized minor extracellular proteases [70]. With new proteases being identified, researchers further constructed WB700 (WB600Δ*vpr*) and WB800 (WB600Δ*wprA*) [71,72]. On the other hand, since the discovery that knocking out endogenous proteases can significantly increase the expression level of secreted proteins, these engineered strains have continuously served as advantageous host strains [70]. Yuan expressed PGA in DB104, detecting an expression level 5 times higher than the wild-type host harboring the same plasmid [73]. This protease-deficient host can also express high levels of endoxylanase [74]. Shi obtained a high-yielding strain of recombinant keratinase using WB600 as a host, while Mu enhanced the expression of d-allulose 3-epimerase [75,76]. Zhang efficiently synthesized CAM-W using WB700 [77]. Zhu achieved high-efficiency expression of nattokinase using WB800 as a host [71]. In summary, the CRISPR-Cpf1 gene editing technology constructed in this study is not only an effective tool for researching endogenous proteases in *Bacillus* but also can be used for constructing high-yield protein host strains.

## Notes

The authors declare no competing financial interest.

## CRediT authorship contribution statement

**Suxin Liu:** Writing – original draft, Data curation, Investigation, Methodology, Visualization. **Fengxu Xiao:** Supervision, Conceptualization, Investigation, Methodology, Visualization. **Youran Li:** Supervision, Conceptualization, Investigation, Methodology, Visualization, Writing – review & editing. **Yupeng Zhang:** Conceptualization, Software, Supervision. **Yanling Wang:** Conceptualization, Methodology, Software. **Guiyang Shi:** Funding acquisition, Supervision.

## Declaration of competing interest

The authors declare no competing financial interest.
