# Supplementary material for: Establishment of the CRISPR-Cpf1 gene editing system in Bacillus licheniformis and multiplexed gene knockout
Source: Synth Syst Biotechnol. 2024 Aug 8;10(1):39–48. doi: 10.1016/j.synbio.2024.08.002 (PMC11366866; doi:10.1016/j.synbio.2024.08.002)
Supplement: Multimedia component 1 [file mmc1.docx]

Supporting information

**Establishment of the CRISPR-Cpf1 gene editing system in *Bacillus licheniformis* and multiplexed gene knockout**

Suxin Liu^1,2,3^, Fengxu Xiao^1,2,3^, Youran Li^1,2,3*^, Yupeng Zhang^1,2,3^, Yanling Wang^1,2,3^, Guiyang Shi^1,2,3^

1. Key Laboratory of Industrial Biotechnology, Ministry of Education, School of Biotechnology, Jiangnan University, Wuxi 214122, People’s Republic of China.

2. National Engineering Research Center for Cereal Fermentation and Food Biomanufacturing, Jiangnan University, 1800 Lihu Avenue, Wuxi 214122, Jiangsu, People’s Republic of China

3. Jiangsu Provincial Engineering Research Center for Bioactive Product Processing, Jiangnan University, Wuxi 214122, Jiangsu, People’s Republic of China.

*Corresponding authors:

Youran Li

E-mail: liyouran@jiangnan.edu.cn

**Construction process of xylose-induced mCherry protein plasmid**

In order to construct the xylose-inducible plasmid, firstly, the xylose promoter fragment was amplified from the genome of the original bacterium of *Bacillus licheniformis* by fragment PCR using primer pair P*_xyl_*-F and P*_xyl_*-R. Then, the vector pHY-P*_2_*-mCherry kept in our laboratory was double digested with two restriction endonucleases, *Hin*dIII and *Kpn*I, and after homologous recombination, the recombinant plasmid induced by xylose to express mCherry protein was finally obtained.

Below, we provide a simple schematic of the constructed plasmid, accompanied by a plasmid digestion validation diagram to give you a clearer picture of the whole process **(Fig. S1)**. The primers required are shown in **Table 1**.


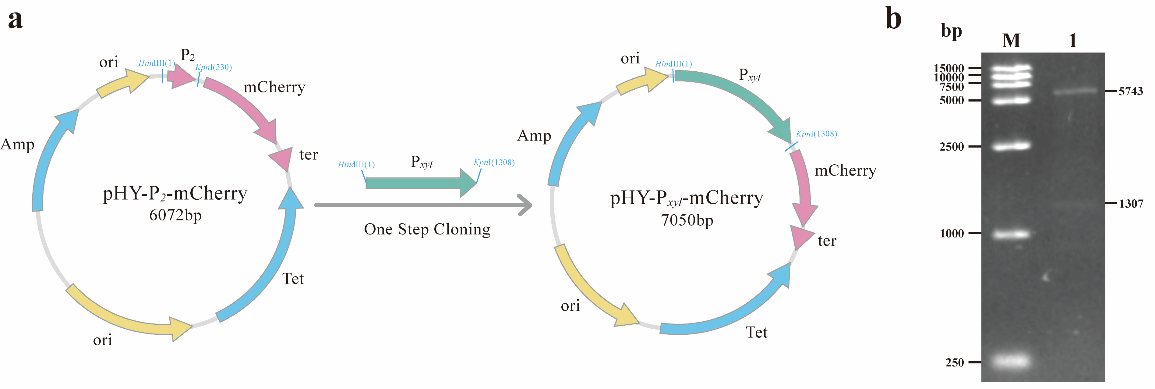


**Fig. S1 Plasmid construction and restriction enzyme digestion verification.** (a) Plasmid pHY-P*_xyl_*-mCherry construction strategy. (b) Plasmid pHY-P*_xyl_*-mCherry restriction enzyme digestion verification; M: DL15000 DNA Marker.

**Table 1 Primers used for plasmid pHY-P*_xyl_*-mCherry in this study**

| **Primers** | **Sequences (5′-3′)** | **Purpose** |
| --- | --- | --- |
| P*_xyl_*-F | aaaacgctttgcccAAGCTTTTAAAATCTCTCATTCATAAACCGTTCCAGAAAATGC | P*_xyl_* gene sequence |
| P*_xyl_*-R | cccttgctcaccatGGTACCTAAACTAACTTCCTTGTATTTATTTTACAAGATGAATCAATCGC |  |

**Comparison of maltose promoters and xylose promoters using mCherry protein as a reporter gene**

We used two recombinant strains of *Bacillus licheniformis* (induced mCherry with maltose and xylose, respectively) as test strains to examine the differences in the fluorescence values produced after the addition of different inducers, and the results obtained are shown below. The left graph shows the data related to the previously tested strain with maltose-induced mCherry protein (**Fig. S2a**), and the right graph shows the data of the strain with xylose-induced mCherry protein (**Fig. S2b**).

Overall, maltose is the most efficient inducer of the maltose promoter, while the different carbon sources all show some strength of induction for the xylose promoter, although xylose is a bit stronger than the rest, but the difference is not significant enough, so the comparison between the two seems to be that the maltose promoter possesses a higher degree of rigour.

**
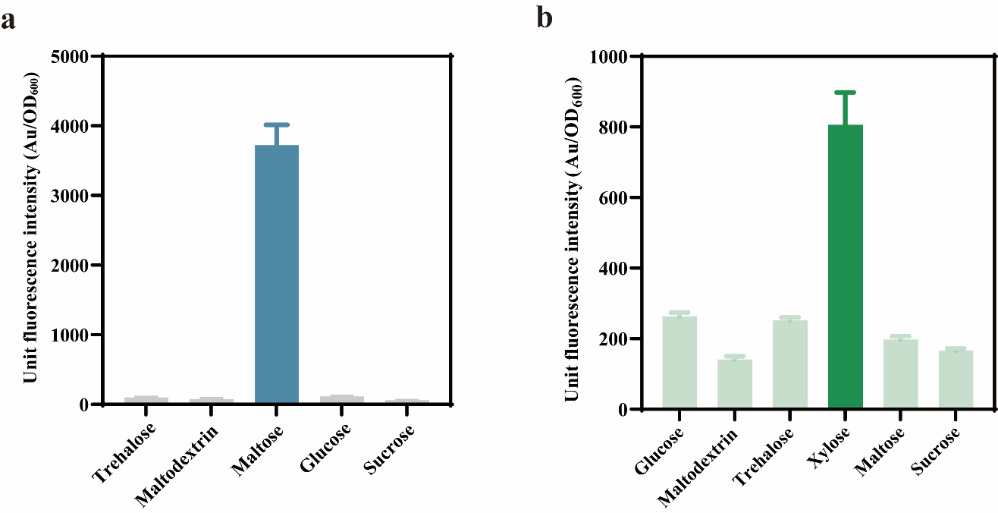
**

**Fig. S2 Expression of mCherry under the control of promoter P*_mal_* and P*_xyl_*.** (a) Effect of different carbon sources on the strength of promoter P*_mal_* in the mCherry reporter assay. (b) Effect of different carbon sources on the strength of promoter P*_xyl_* in the mCherry reporter assay.

**Validation of maltose-inducible promoters using mTagBFP2 and trehalase as reporter genes**

Subsequently, we further tested the strength of the maltose promoter using two proteins, mTagBFP2 and trehalase, respectively, as reporter genes, and the two reporter genes were detected as shown below:

The blue fluorescent protein was detected by diluting the sample to an OD_600_ value of 0.5-2.0, adding 200 μL of the sample to a 96-well plate and placing it in Microplate Reader for detection, the detection method was as follows: the detection area was selected, and the enzyme plate was shaken for 10 s, the excitation wavelength was 400 nm, and the absorption light was 450 nm, the gain value was 141. The fluorescence value of the sample (AU/OD_600_) =fluorescence value of the experimental group−control (recombinant strain carrying pHY-PLK300 plasmid) fluorescence value.

The enzyme activity (trehalase) was determined by the following protocol: 1 mL of fermentation broth was taken into a 1.5 mL Ep tube and centrifuged at 12000 rpm for 10 min at 4°C using a refrigerated centrifuge, and the supernatant was taken as the crude enzyme solution. 980 µL of reaction solution (10 mM pPNG, 100 mM Kcl, 50 mM Hepes-NaOH buffer, pH 8.0) was added to 20 µL of the crude enzyme solution, and the reaction was carried out at 30°C for 10 min. Afterwards, the reaction was stopped by boiling for 5 min, and 200 µL of the reaction solution was added to a 96-well plate, and the absorbance at 410 nm was measured by Microplate Reader. Based on the standard curve, the content of reducing sugars was calculated, and the enzyme activity was calculated according to the enzyme activity formula. One unit of enzyme activity (U) was defined as the amount of enzyme required to release 1 µmoL of p-nitroaniline per hour at 30°C, pH 8.0.

First, we screened to obtain the *Bacillus licheniformis* maltose-inducible elements using mTagBFP2 as a reporter gene. The three promoters to be tested, P*_mal_* (nucleotide sequence as shown in SEQ ID NO.1), P*_mdxD_* (nucleotide sequence as shown in SEQ ID NO.2), and P*_amyL_* (nucleotide sequence as shown in SEQ ID NO.3), were the promoters derived from *Bacillus licheniformis* maltose manipulator, promoter of β-amylase gene, and promoter of ɑ-amylase gene, respectively. Each of the three promoters to be tested was assembled and ligated into the pHY-PLK300 plasmid with the mTagBFP2 (blue fluorescent protein) gene in vitro by overlap extension PCR, corresponding to the formation of three recombinant plasmids, respectively pHY-P*_mal_*-mTagBFP2, pHY-P*_mdxD_*-mTagBFP2, and pHY-P*_amyL_*-mTagBFP2. The above three plasmids were transformed into the receptor cells of *B. licheniformis* to form the recombinant strains M1, M2, M3. The three recombinant strains were cultured in the fermentation medium under two conditions (−. no maltose; +. 1.5% maltose) and the fluorescence value was measured for 24 h.

The results are shown in **Fig. S3**, M1 strain did not detect fluorescence value when no maltose was added, and the fluorescence value was 583 AU/OD_600_ when maltose was added. M2 strain showed fluorescence value of 697 AU/OD_600_ when no maltose was added, and the fluorescence value was 3068 AU/OD_600_ when maltose was added. M3 strain showed fluorescence of 557 AU/OD_600_ when no maltose was added, and the fluorescence value was 393 AU/OD_600_. M1 and M2 strains showed significant maltose induction properties and M1 strain showed strict induction of maltose. Therefore, P*_mal_* was selected as the maltose-inducible element to control the expression of genes for Cpf1.


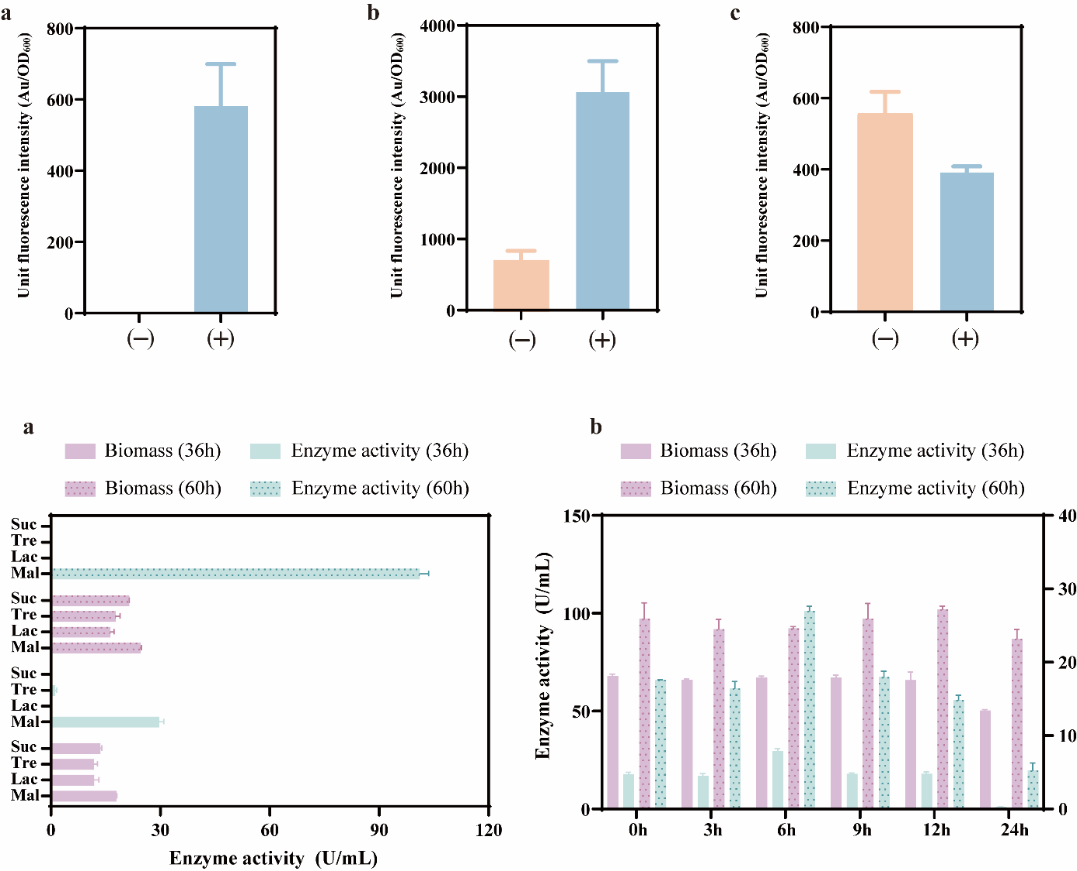


**Fig. S3 Screening of *Bacillus licheniformis* for inducible elements using mTagBFP2 as a reporter gene** (a) Effect of the addition of 1.5% maltose or not on the fluorescence intensity of the recombinant strains M1. (b) Effect of the addition of 1.5% maltose or not on the fluorescence intensity of the recombinant strains M2. (c) Effect of the addition of 1.5% maltose or not on the fluorescence intensity of the recombinant strains M3.

Secondly, the optimal induced expression conditions of the P*_mal_* promoter were determined with the aid of trehalase. The promoter P*_mal_* was assembled and ligated into pHY-PLK300 plasmid by overlap extension PCR in vitro with *Bacillus subtilis* *sacB* signal peptide, endogenous trehalase gene of *Bacillus licheniformis*, and amyL terminator (P*_mal_*+ *sacB* signal peptide+ trehalase+ terminator sequences as shown in SEQ ID NO.4), and the recombinant plasmid was electroporated into *Bacillus licheniformis* to construct the recombinant strain MA. The parameters of the recombinant strain MA were tested under different inducers (maltose, trehalose, sucrose, lactose), and the time of inducer addition, respectively. The results are shown in **Fig. S4**, which indicate that 6 h addition of 1.5 % maltose has the best induction effect. This provides an optional control scheme for CRISPR-Cpf1-mediated gene editing technology.


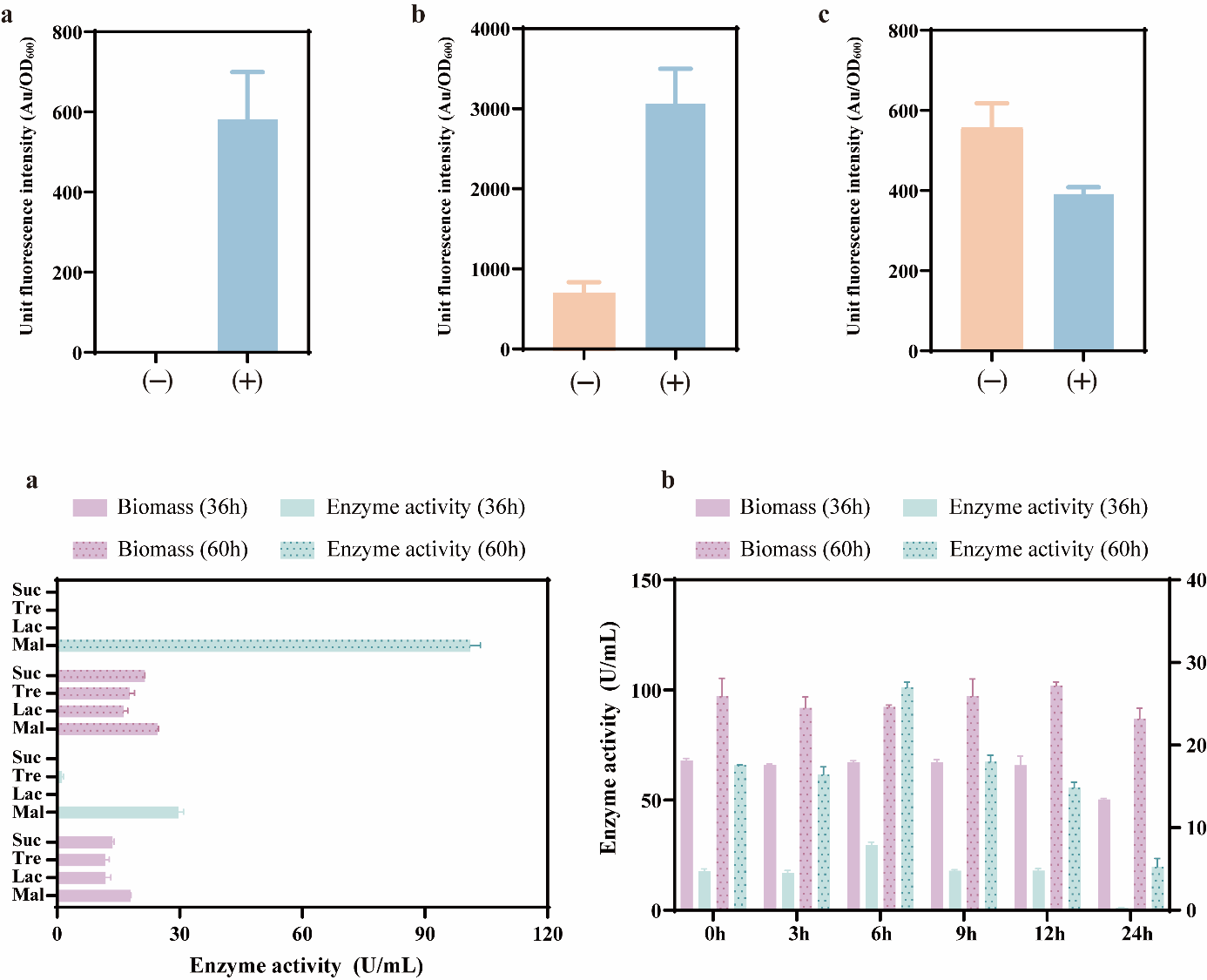


**Fig. S4 Optimal conditions for induced expression of the Pmal promoter were determined with the aid of trehalase.** (a) Effect of the addition of different carbon source inducers on the enzyme activity of trehalase and the biomass of *Bacillus licheniformis*. (b) Effect of time of addition of 1.5% maltose inducer on enzyme activity of trehalase and biomass of *Bacillus licheniformis*.


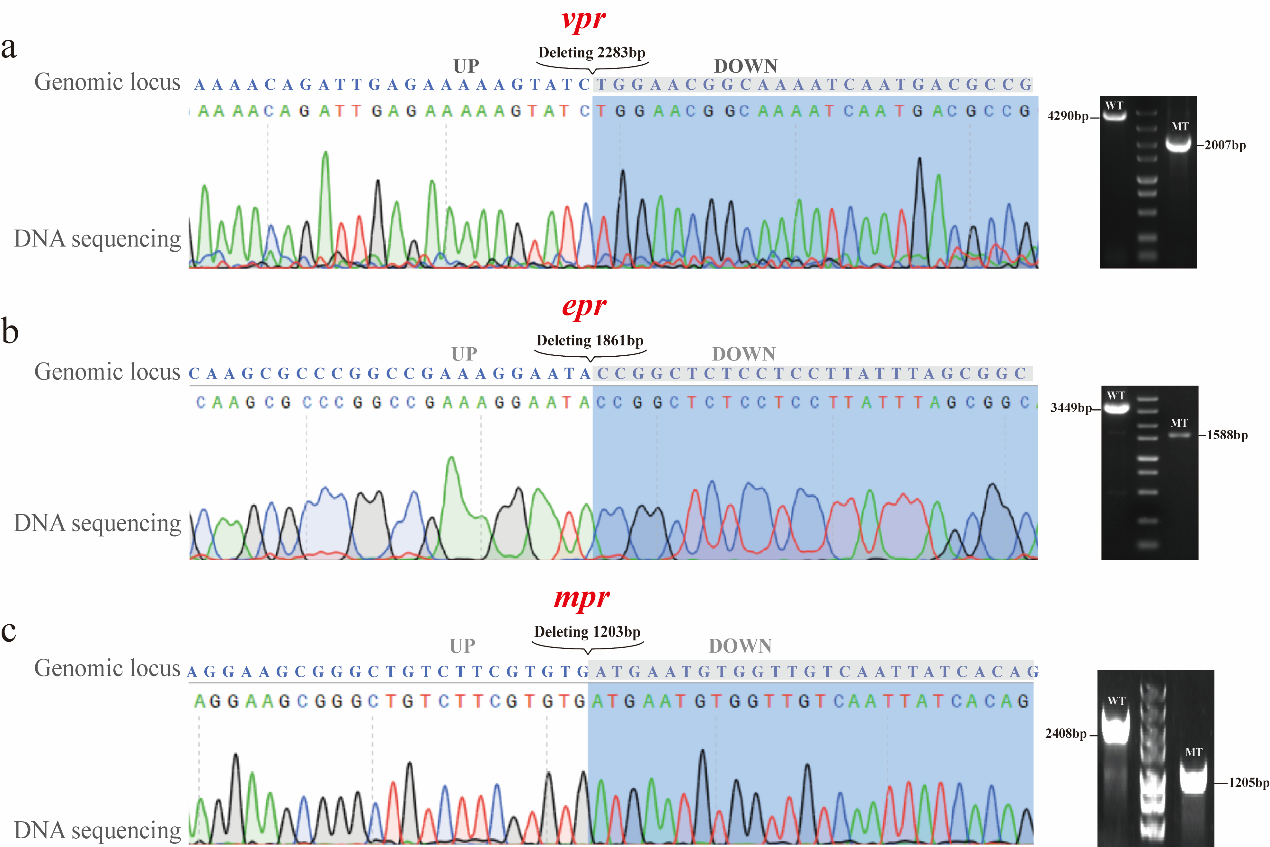


**Fig. S5 Sequencing graphs of the three knockout strains**

**
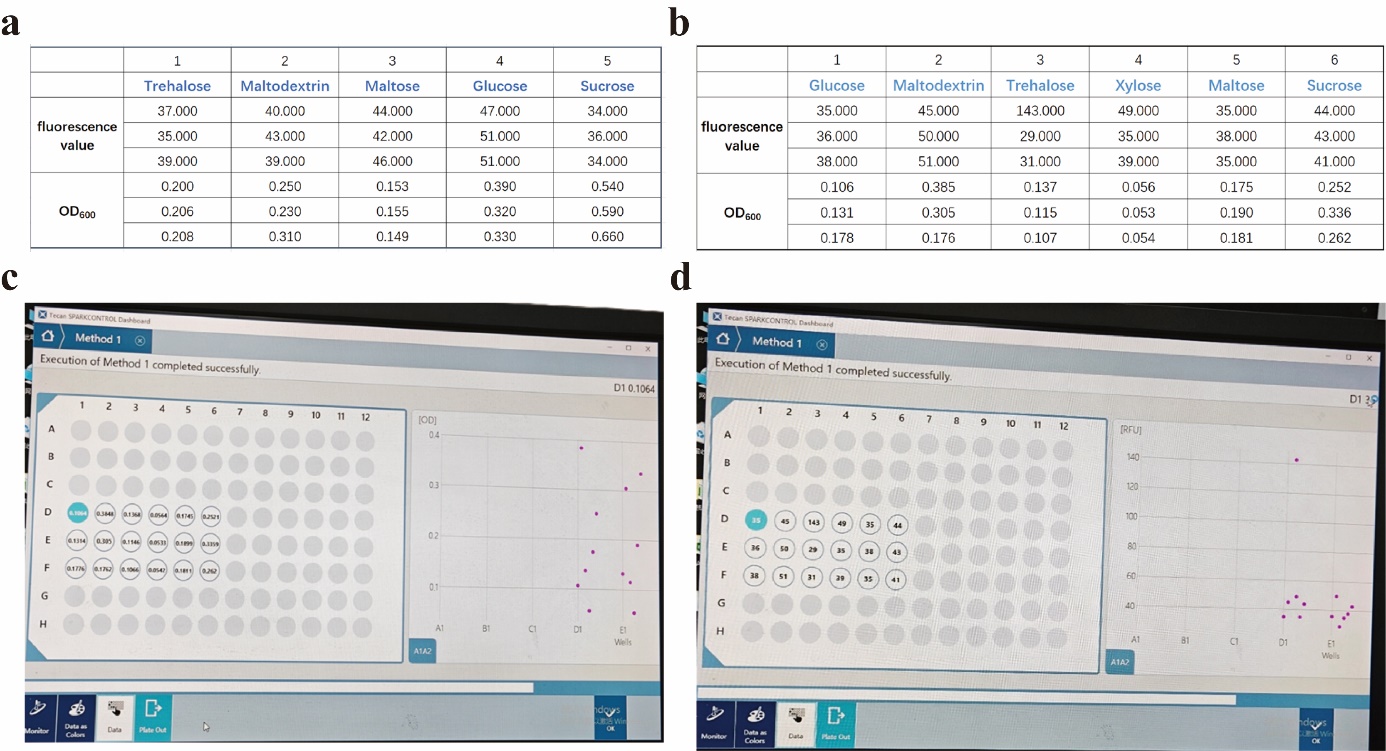
**

**Fig. S6 The fluorescence photos of the raw data** (a) Data for fluorescence values and biomass of recombinant strains of mCherry protein induced by maltose. (b) Data for fluorescence values and biomass for xylose-induced mCherry protein recombinant strains. (c) Data picture of the biomass displayed on the Microplate Reader for xylose-induced mCherry protein recombinant strains. (d) Data picture of the fluorescence values displayed on the Microplate Reader for xylose-induced mCherry protein recombinant strains.

**
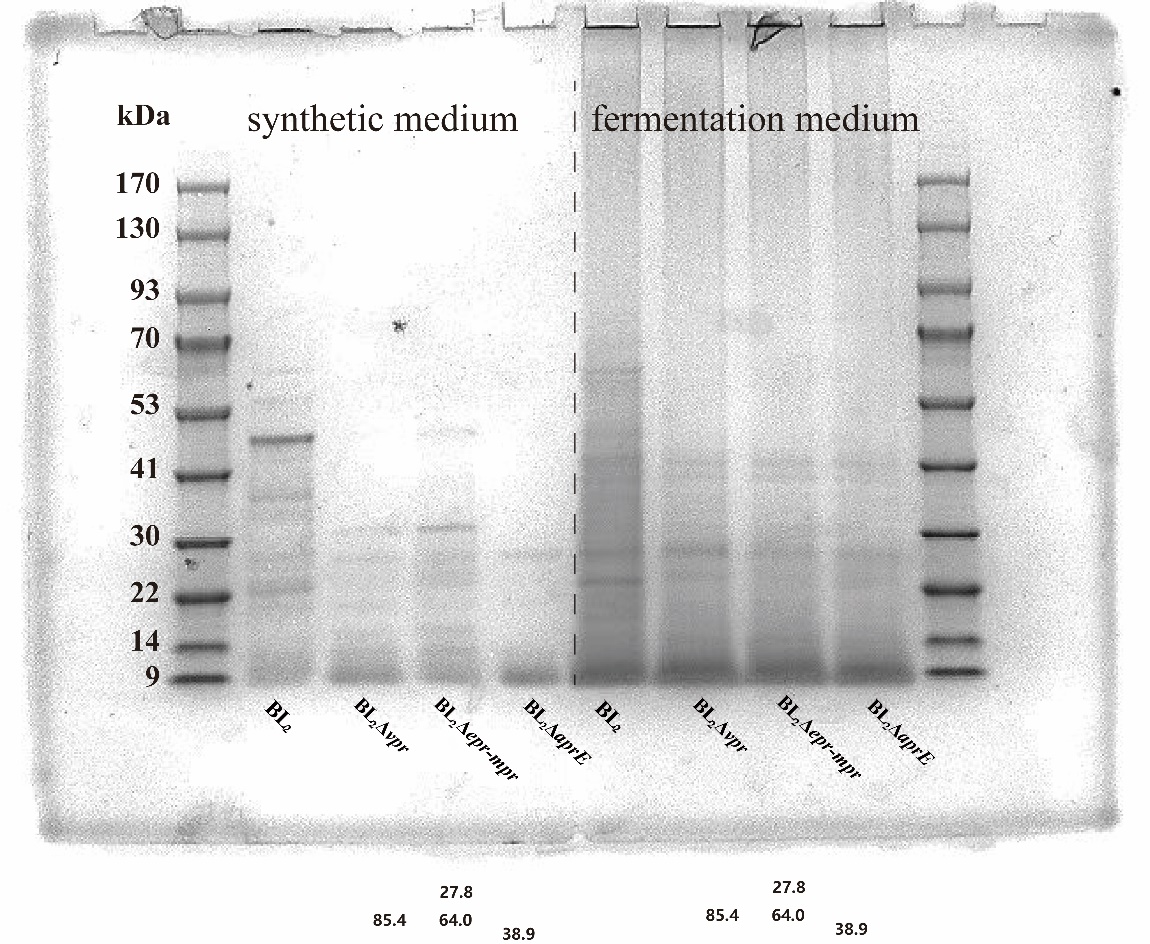
**

**Fig. S7 The extracellular protein gel image**


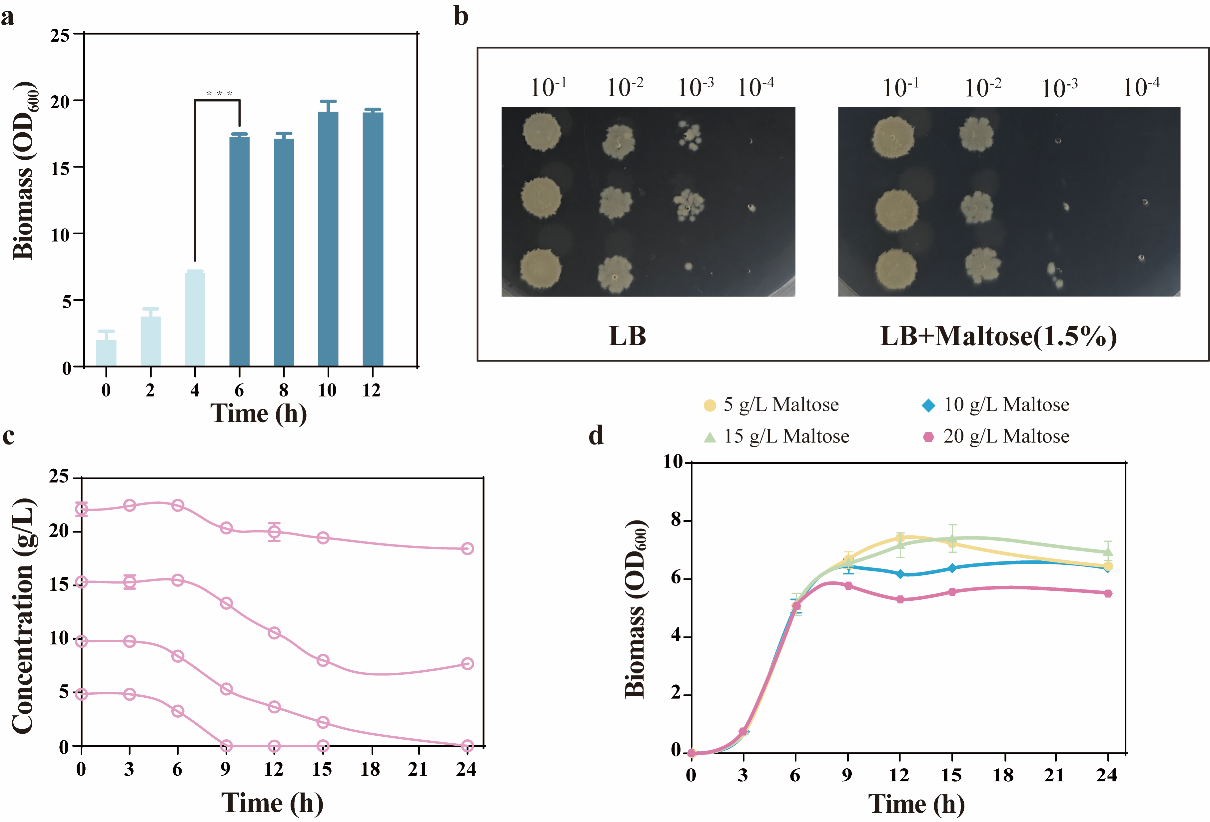


**Fig. S8: The growth status of the strain under different maltose induced conditions.** (a) Effect of adding maltose at different times on biomass. (b) a spot assay test was carried out on LB and LB plates containing 1.5% maltose. (c) Effect on maltose consumption by *Bacillus licheniformis* wild bacteria of adding different concentrations of maltose at 0 h (d) Effect of adding different concentrations of maltose at 0 h on the biomass of *Bacillus licheniformis* wild bacteria.


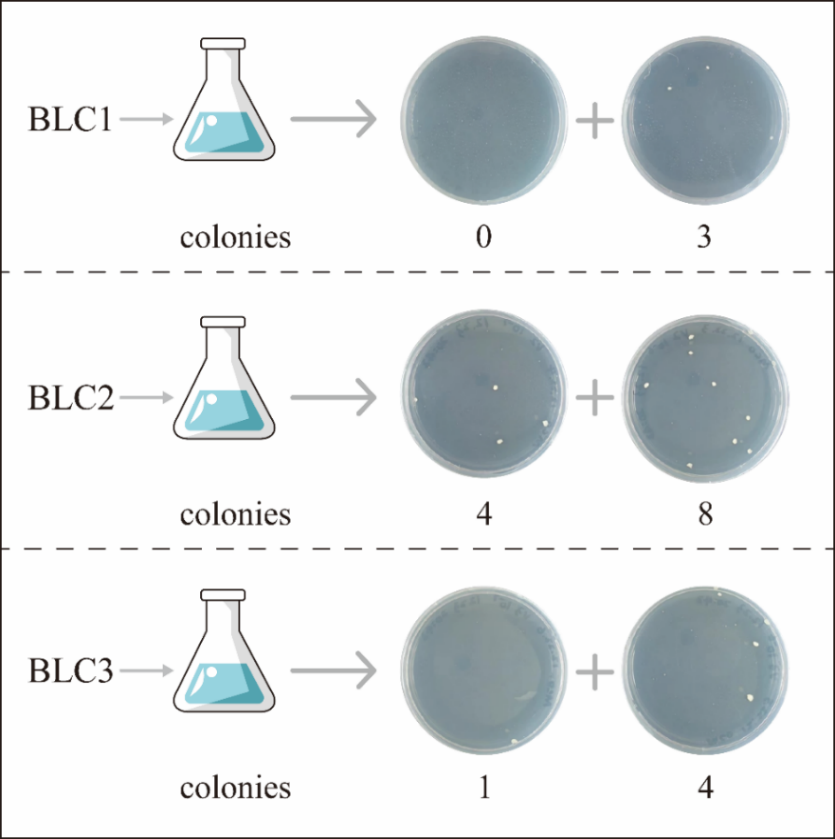


**Fig. S9 Statistical table of colony number**

**
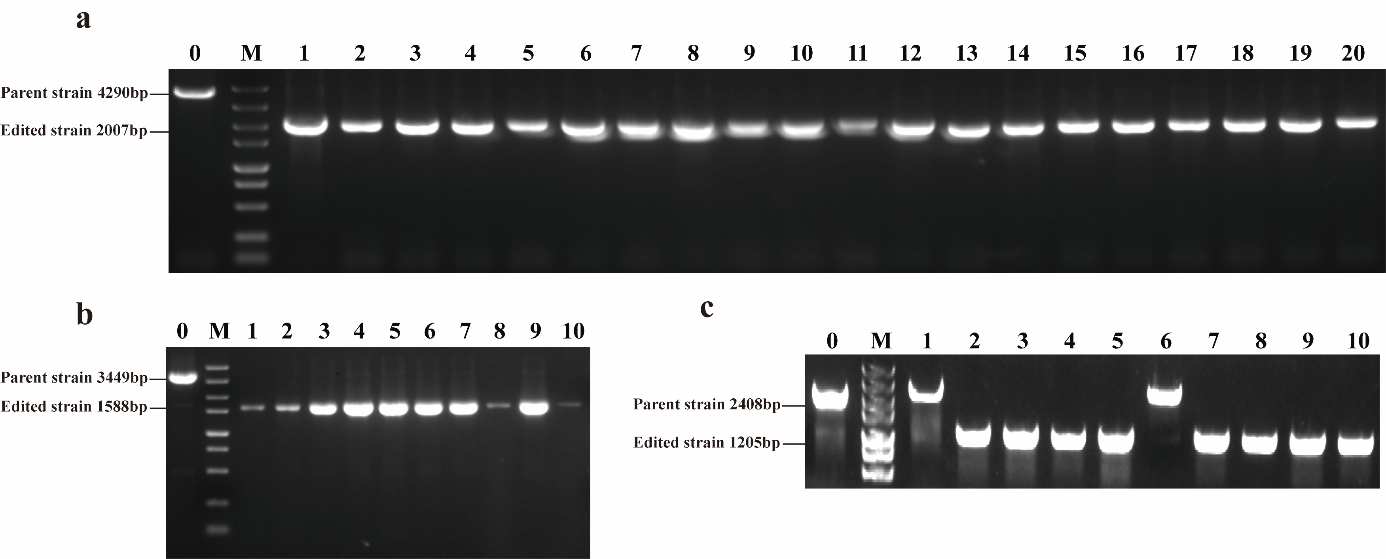
**

**Fig. S10 Evaluation of the efficiency of the CRISPR-Cpf1 system in *Bacillus licheniformis*.** (a) Disruption of the *vpr* gene, 1~20 are PCR amplification results of positive monoclonal colonies; M: DL5000 DNA marker. (b)Disruption of the *epr* gene, 1~10 are PCR amplification results of positive monoclonal colonies; M: DL5000 DNA marker. (c)Disruption of the *mpr* gene, 1~10 are PCR amplification results of the same positive monoclonal colonies as described above; M: DL5000 DNA marker.


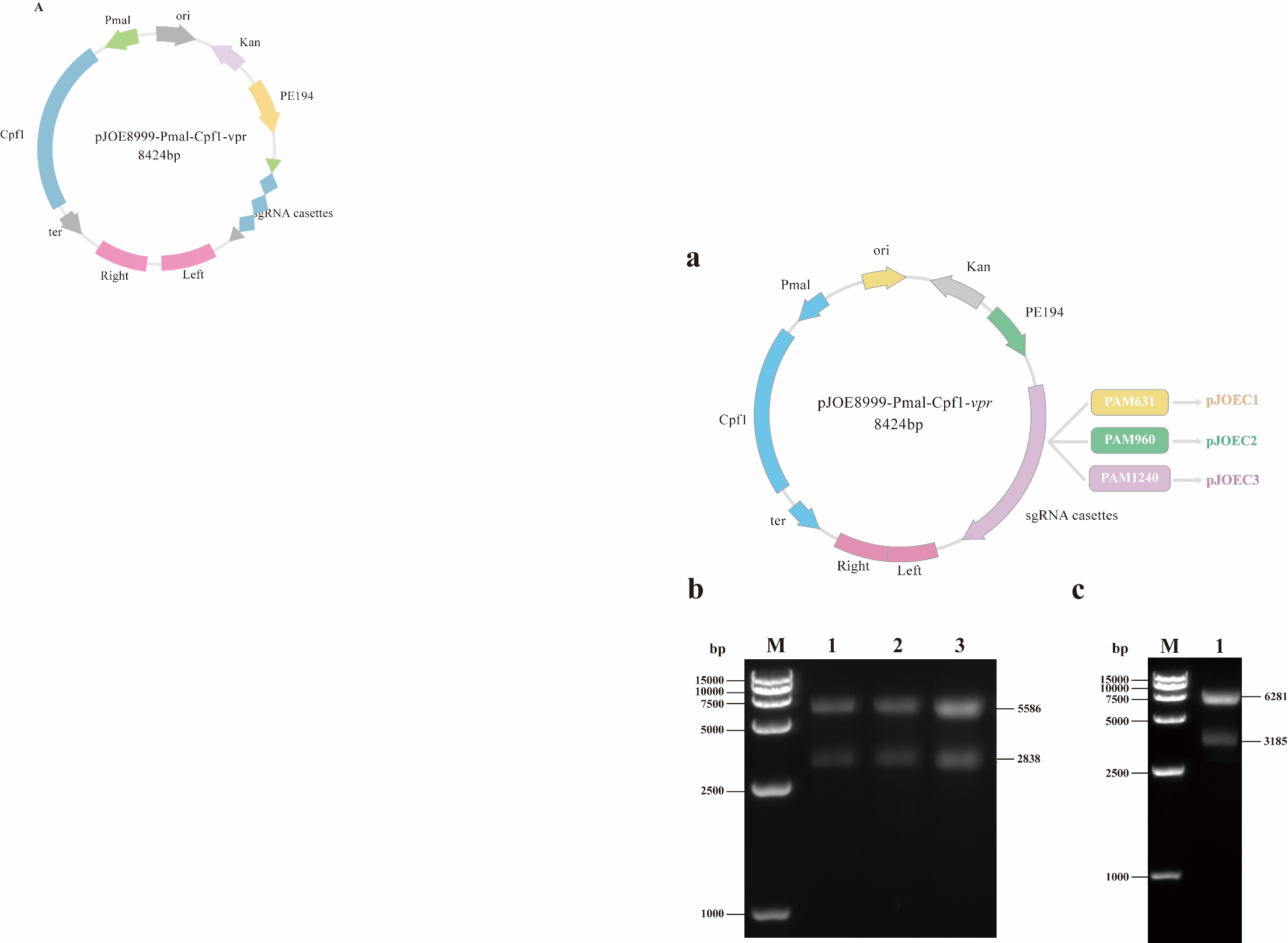


**Fig. S11 The knockout plasmids** (a)The *vpr* knockout plasmids (three knockout plasmids were obtained by selecting three different targets, named pJOEC1, pJOEC2, pJOEC3). (b) Digest validation of the three recombinant plasmids used for *vpr* knockout; M: DL15000 DNA Marker. (c) Digest validation of recombinant plasmids used for simultaneous knockout of both *epr* and *mpr* genes; M: DL15000 DNA Marker.


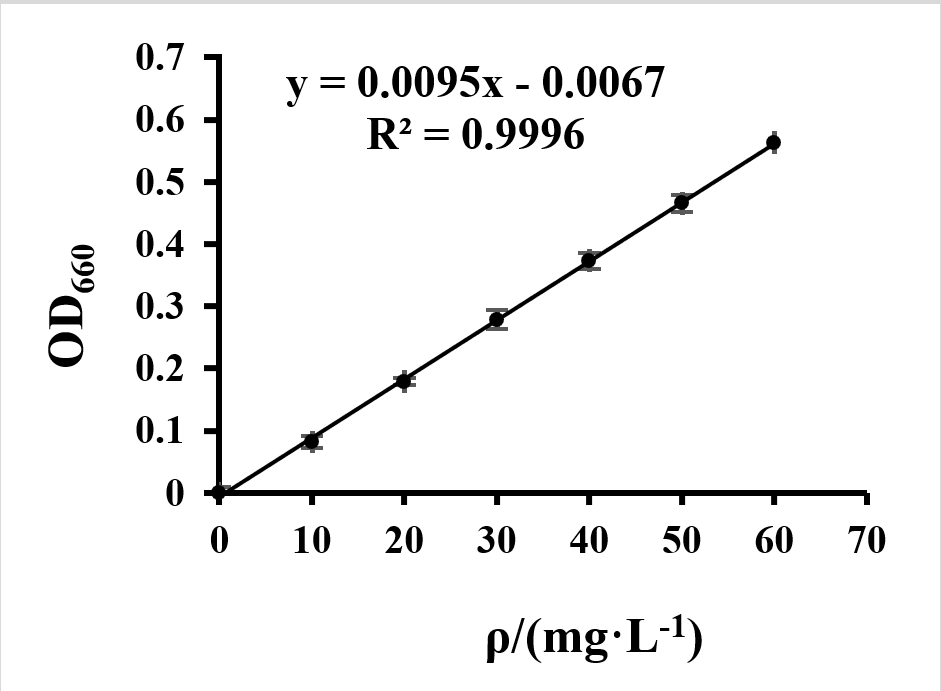


**Fig. S12 The standard curve of tyrosine.**

**
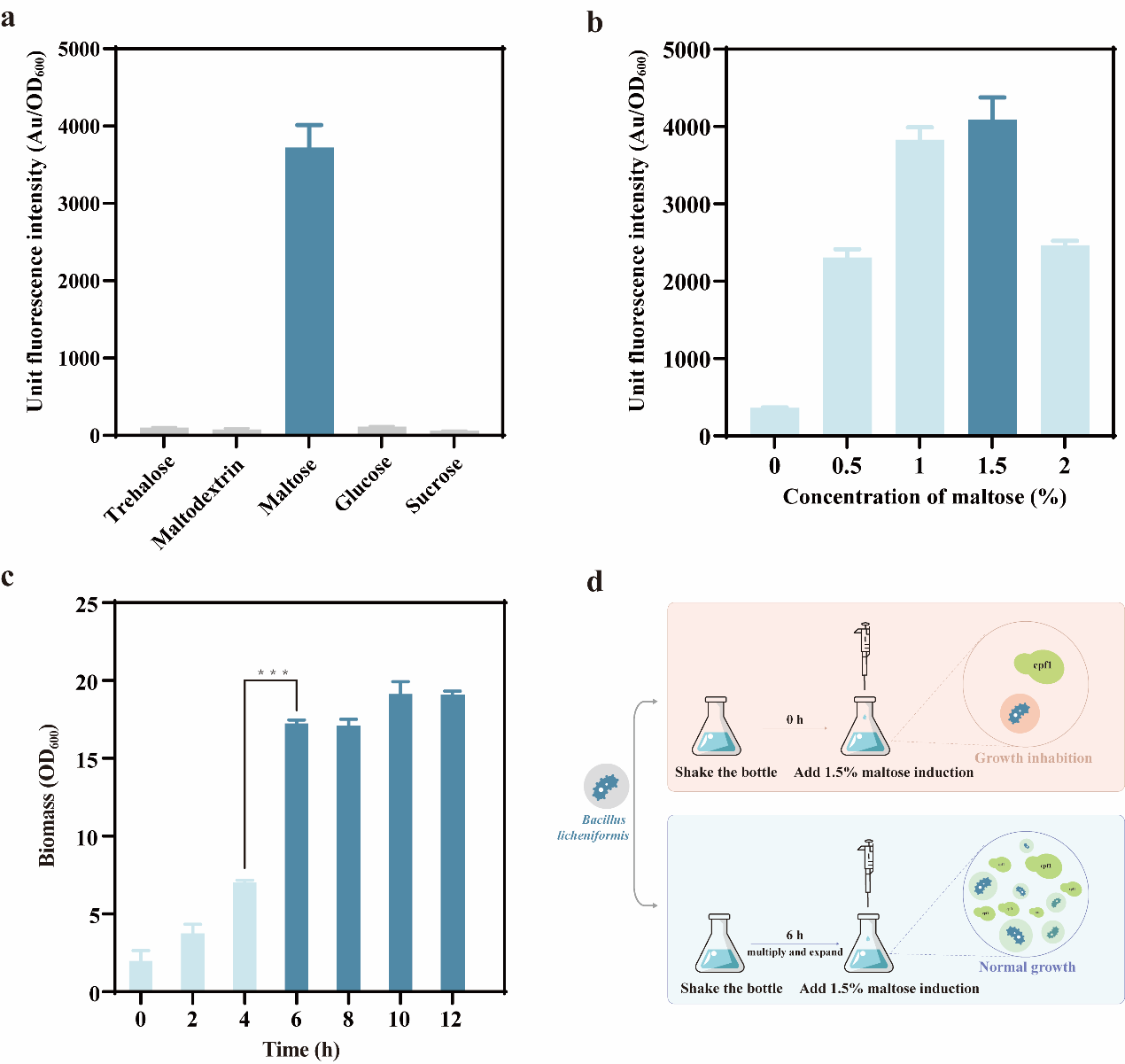
**

**Figure 2. Expression of mCherry under the control of mal promoter.** (a) Effect of different carbon sources on the strength of the mal promoter in the mCherry reporter assay. (b) Effect of different concentrations of maltose on the strength of the mal promoter in the mCherry reporter assay. (c) The growth status of the strain under different maltose induced conditions (d) Strain growth was inhibited by adding maltose at 0 h and normalized by adding maltose at 6 h.

SEQ ID NO.1

Actccaattcagtgaattgcctttattctacacgacataaaacggccgggaaagttcccgtttttcgggaaaataaacagaacgcgagtataggaactgtctttcccgaacctgttggaacggctccttcagcatgatataagtaaattgtaaacgcttataagggggctttaaaaa

SEQ ID NO.2

agggtctcattctgttaccggtaacaagctgaaaatgattgttcctgttatgccgtcatgataatttcagaataaaagccggtttatcacagccggacaaccaaaaagggggaaac

SEQ ID NO.3

Aataaatgagaagaaagcgccatatcggcgcttttctgttgcaagaaaatatagggaaaacgatatttgctaaaaattccaaatatttatacaatagcatgtgtttcactttgaaaggggagaggaaaatc

SEQ ID NO.4

actccaattcagtgaattgcctttattctacacgacataaaacggccgggaaagttcccgtttttcgggaaaataaacagaacgcgagtataggaactgtctttcccgaacctgttggaacggctccttcagcatgatataagtaaattgtaaacgcttataagggggctttaaaaacTCGAGATGAACATCAAAAAGTTTGCAAAACAAGCAACAGTATTAACCTTTACTACCGCACTGCTGGCAGGAGGCGCAACTCAAGCGTTTGCGCATCATCATCATCATCATGGTACCatggaaacgaaagaaaatccatggtggaaaaaagcagtcgtctatcagatttatccgaaaagctttaaagatacaacaggtaacggtgtcggcgacattcgcggcatcatcgaaaagctcgactacatcaaggagctcgcctgtgatgtcatttggctgacgccgatttatcaatcgccgcaaaatgacaacggctatgacatcagcgactattacaacattcatgaagaatatggaacaatggccgactttgaagagcttctagaagaggctcacaaacgcggcataaaggtgatcatggatcttgtcgtcaaccatacatcaacagagcacaaatggtttaaagaagcagcgtccggaaaagaaaatccgtatcgcgacttttatatttggaaggacatgaagccggacggtgccccgccgacaaactgggaatcgaagttcggcggttcggcgtgggagtttcatgaagagaccggacagtattatctccatctctatgatgtgacacaggcggatttgaattgggagaacgatgaagtcagaaaaaaagtgtacgagatgatgcatttctggtttgaaaaaggaatcgacggcttcagacttgatgtcatcaatgtcatctcaaaagaccagcgtttccctgatgatgacgagggggacgggcgcagattttatacggacgggccgagggttcacgagtttttgaatgaaatgaacagggaggtcttttcaaaatacgacagcatgacggtcggggaaatgtcatcgacgacaatcgcagattgcatccggtatacaaaccctgaaagccgggagctcgatatggtgtttaactttcaccatttaaaggccgactatccaaacggggaaaaatgggcgctggcggattttgattttctaaagctgaagaagattctttctgaatggcagactgaaatgcataaaggaggcgggtggaacgcattgttctggtgcaaccacgaccagccgcggatcgtctcacgctacggagacgacggaaaataccgcataaaatctgcgaaaatgctggcaaccgcaattcatatgcttcagggaacgccatacatttaccagggagaagagctcgggatgaccaacccgaaattcgatgacatctctctttatcgggatgtcgaatctctcaatatgtaccgtctgctgaaagaagcaggaaagccggaagccgaaatcatcgaaatcctgaaagcgaagtcccgtgataattcaaggacgccggtgcagtggaacggagaaaaaaatgccgggtttacgacggggacaccgtggatttcggtgcctgccaactacaaagaaatcaacgcagaaaaagcgctcagcgatcctgactcgattttctaccattataaaaagctgaataagctccgcaaggaattcgatatcatcacaaccggagattatcagctgattctggaagacgatcaagcactttatgcgtatttgagaaacggagcggatgaaaaactgctggtgatcaataatttttacgggaaagagaccgagtttcagctccctgatgatattgatattgacggctatgatgccaaggttctcatttccaatgacaccgatcttcccgagtcatttacacgctttaaagtgaaaccgtatcaatcgattgtttatcatctcgccaaaccatgctaaGTCGACATTCAGCCAGGCGGATTTTTCATTTTGGGGGCGGTCTAACAGGATAAGCTCCAGATCCTGCTATCAATACCAAGTCACTGAATTACCCGTCATGATTCCTTTCCTATTGCTTGTTGTTATGACGGGTAACTTCTATAATTAGGATTTATTTAGAGTGAATGGTTTTTTACCC
