# Supplementary material for: Establishment of the CRISPR-Cpf1 gene editing system in Bacillus licheniformis and multiplexed gene knockout
Source: Synth Syst Biotechnol. 2024 Aug 8;10(1):39–48. doi: 10.1016/j.synbio.2024.08.002 (PMC11366866; doi:10.1016/j.synbio.2024.08.002)
Supplement: Multimedia component 2 [file mmc2.docx]

**Table S1 Bacterial strains, plasmids used in this study**

| **Name** | **Relevant genotype** | **Reference** |  |
| --- | --- | --- | --- |
| **Strains** |  |  |  |
| *Escherichia coli* JM109 | *F', traD36, proABΔ.lacIq, Δ(lacZ), M15/Δ(lac-proAB), glnV44, e14−, gyrA96, recA1, relA1,endA1, thi, hsdR17* | Our lab |  |
| *Bacillus licheniformis* CICIM B1391 | Wild-type | Our lab |  |
| *Bacillus subtilis* 168 | Wild-type | Our lab |  |
| BLP | B. licheniformis CICIM B1391 harboring plasmid pHY | This work |  |
| BLPM | B. licheniformis CICIM B1391 harboring plasmid pHY-P*_mal_*-mCherry | This work |  |
| BLC1 | B. licheniformis CICIM B1391 harboring plasmid pJOEC1 | This work |  |
| BLC2 | B. licheniformis CICIM B1391 harboring plasmid pJOEC2 | This work |  |
| BLC3 | B. licheniformis CICIM B1391 harboring plasmid pJOEC3 | This work |  |
| BLCME | B. licheniformis CICIM B1391 harboring plasmid pJOECME | This work |  |
| BL_2_∆*vpr* | B. licheniformis CICIM B1391, ∆*vpr* | This work |  |
| BL_2_∆*aprE* | B. licheniformis CICIM B1391, ∆*aprE* | Our lab |  |
| BL_2_∆*epr-mpr* | B. licheniformis CICIM B1391, ∆*epr*, ∆ *mpr* | This work |  |
| **Plasmids** |  |  |  |
| pHY | *E. coli*/*Bacillus* shuttle vector, Ap^R^/Tet^R^ | Our lab | |
| pHY-eGFP | pHY, with the eGFP cassette | Our lab | |
| pHY-P*_mal_*-eGFP | pHY, with the eGFP cassette mediated by P*_mal_* promoter | This work | |
| pHY-P*_mal_*-mCherry | pHY, with the mCherry cassette mediated by P*_mal_* promoter | This work | |
| pJOE8999 | *E. coli*/*Bacillus* shuttle vector, Kan^R^ | Our lab | |
| pJOEC1 | pJOE8999 derivative with Cpf1 targeting the PAM631 locus of *vpr* | This work | |
| pJOEC2 | pJOE8999 derivative with Cpf1 targeting the PAM960 locus of *vpr* | This work | |
| pJOEC3 | pJOE8999 derivative with Cpf1 targeting the PAM1240 locus of *vpr* | This work | |
| pJOECEM | pJOE8999 derivative with Cpf1 targeting the locus of *epr* and *mpr* | This work | |

Ap^R^, ampicillin resistance; Tet^R^, tetracycline resistance; Kan^R^, kanamycin resistance

**Table S2** **Primers used in this study**

| **Primers** | **Sequences (5′-3′)** | **Purpose** |  |
| --- | --- | --- | --- |
| P*_mal_*-*Bgl*II-F | GGAAGATCTactccaattcagtgaattgcctttattctacac | P*_mal_* gene sequence |  |
| P*_mal_*-*Xho*I-R | CCGCTCGAGtttttaaagcccccttataagcgtttacaatttact |  |  |
| mCherry -F | ggctttaaaaacTCGAGatggtgagca | mCherry gene sequence |  |
| mCherry-R | GGATCTGGAGCTTATCCTGTTAGACCG |  |  |
| P*_mal_*-F | GGTCATGGCCATTCTTCTGTACAACTCCAATTCAGTGAATTGC | P*_mal_* gene sequence | |
| P*_mal_*-R | AACTCTTGGTAGATAGACATCTCGAGTTTTTAAAGCCCCCTTATAA |  |  |
| Cpf1-F | AAGGGGGCTTTTAAAAACTCGAGATGTCTATCTACCAAGAGTT | Cpf1 gene sequence | |
| Cpf1-R | ATCCGCCTGGCTGAATGTCGACTTAGTTGTTACGGTTTTGAA |  |  |
| ter-F | TCAAAACCGTAACAACTAAGTCGACATTCAGCCAGGCGGATTTTT | ter gene sequence | |
| ter-R | GGGTCGACGGCCAACGAGGCCCGGGGGATCCAGGTACCGGGTAAAAAACCATTCACTCT |  |  |
| crRNA-F | TTCCTTTTTGCGTGTGATGCGAATTCTCTTTTCGATTTTTATGAA | crRNA gene sequence | |
| crRNA -R | TTACCCGGTACCTGGATCCCCCGGGTATAAACTTTTCAGTTGCAG |  |  |
| *vpr*-Left-F | CTGCAACTGAAAAGTTTATACCCGGGCATACCGATCCCCTCCAGTT | *vpr*-Left gene sequence | |
| *vpr*-Left-R | TCATTGATTTTGCCGTTCCAGATACTTTTTCTCAATCTGT |  |  |
| *vpr*-Right-F | ACAGATTGAGAAAAAGTATCTGGAACGGCAAAATCAATGA | *vpr*-Right gene sequence | |
| *vpr*-Right-R | TGGTTTTTTACCCGGTACCTGGATCCTCAATTTAGCGTTTTTGCTG |  |  |
| *vpr*-qiaochu-yz -F | agggcgatgtggatatgatcg | Diagnostic PCR | |
| *vpr*-qiaochu-yz -R | gccagactttacaaagagaggttcgaac |  |  |
| *mpr*-*epr*-crRNA-F1 | TTTTGCGTGTGATGCGAATTCTCTTTTCGATTTTTATGAAACAATTCAACCTTAAATTT | *epr*-*epr*-crRNA gene sequence | |
| *mpr*-*epr*-crRNA-R1 | aaagagatcacacagccgttggaATCTACAACAGTAGAAATTAAATGCTCCTTATATAAACAAAAGT |  |  |
| *mpr*-*epr*-crRNA-F2 | tccaacggctgtgtgatctctttAATTTCTACTGTTGTAGAT |  |  |
| *mpr*-*epr*-crRNA-R2 | attcaggaccgattgccatctccATCTACAACAGTAGAAATT |  |  |
| *mpr*-*epr*-crRNA-F3 | ggagatggcaatcggtcctgaatAATTTCTACTGTTGTAGAT |  |  |
| *mpr*-*epr*-crRNA-R3 | aaaacggcatttttgCCCGGGTATAAACTTTTCAGTTGCAGA |  |  |
| *epr*-Left-F | AAGTTTATACCCGGGcaaaaatgccgttttcattgaatataaagtagaaggc | *epr*-Left gene sequence | |
| *epr*-Left-R | aaggaggagagccggtattcctttcggccgggcg |  |  |
| *epr*-Right-F | cggccgaaaggaataccggctctcctccttatttagcg | *epr*-Right gene sequence | |
| *epr*-Right-R | ggtgttgtatccgagtagctacccaccctaaacacaatgcaa |  |  |
| *mpr*-Left-F | tagggtgggtagctactcggatacaacaccatacgataaccg | *mpr*-Left gene sequence | |
| *mpr*-Left-R | gacaaccacattcatcacacgaagacagcccgc |  |  |
| *mpr*-Right-F | ggctgtcttcgtgtgatgaatgtggttgtcaattatcacagcg | *mpr*-Right gene sequence | |
| *mpr*-Right-R | CCGGTACCTGGATCCtcattgctttcggttgcgatcg |  |  |
| *mpr*-qiaochu-yz-F | ggaatcgggggtgtcatgttt | Diagnostic PCR | |
| *mpr*-qiaochu-yz-R | aagccacttgttcaggctcgccaaa |  |  |
| *epr*-qiaochu-yz-F | ATGTTGTGGCAGGACTTTCGAGA | Diagnostic PCR | |
| *epr*-qiaochu-yz-R | TGAACCAGTCGAAATACCAGCCAATA |  |  |
